# Multi-Signal Sedimentation Velocity Analysis with Mass Conservation for Determining the Stoichiometry of Protein Complexes

**DOI:** 10.1371/journal.pone.0062694

**Published:** 2013-05-16

**Authors:** Chad A. Brautigam, Shae B. Padrick, Peter Schuck

**Affiliations:** 1 Department of Biophysics, The University of Texas Southwestern Medical Center, Dallas, Texas, United States of America; 2 Laboratory of Cellular Imaging and Macromolecular Biophysics, National Institute of Biomedical Imaging and Bioengineering, National Institutes of Health, Bethesda, Maryland, United States of America; University of Cambridge, United Kingdom

## Abstract

Multi-signal sedimentation velocity analytical ultracentrifugation (MSSV) is a powerful tool for the determination of the number, stoichiometry, and hydrodynamic shape of reversible protein complexes in two- and three-component systems. In this method, the evolution of sedimentation profiles of macromolecular mixtures is recorded simultaneously using multiple absorbance and refractive index signals and globally transformed into both spectrally and diffusion-deconvoluted component sedimentation coefficient distributions. For reactions with complex lifetimes comparable to the time-scale of sedimentation, MSSV reveals the number and stoichiometry of co-existing complexes. For systems with short complex lifetimes, MSSV reveals the composition of the reaction boundary of the coupled reaction/migration process, which we show here may be used to directly determine an association constant. A prerequisite for MSSV is that the interacting components are spectrally distinguishable, which may be a result, for example, of extrinsic chromophores or of different abundances of aromatic amino acids contributing to the UV absorbance. For interacting components that are spectrally poorly resolved, here we introduce a method for additional regularization of the spectral deconvolution by exploiting approximate knowledge of the total loading concentrations. While this novel mass conservation principle does not discriminate contributions to different species, it can be effectively combined with constraints in the sedimentation coefficient range of uncomplexed species. We show in theory, computer simulations, and experiment, how mass conservation MSSV as implemented in SEDPHAT can enhance or even substitute for the spectral discrimination of components. This should broaden the applicability of MSSV to the analysis of the composition of reversible macromolecular complexes.

## Introduction

The study of protein interactions in multi-component systems is key to improve our understanding of signaling pathways, which ubiquitously possess dynamically assembled multi-protein complexes as critical nodes for integrating different information flows and regulating downstream events. Hallmarks of such complexes are multi-valent interactions and cooperativity, which are notoriously difficult to characterize. We have recently developed a global multi-method analysis for interacting systems with multiple binding sites [1] that is useful for determining thermodynamic parameters of the interactions, including association constants, enthalpy changes, and cooperativity constants. However, often one of the most difficult steps is to identify the thermodynamic states, i.e. to ascertain which complexes exist in solution. This goal can be far from trivial to achieve for two-component interactions and be very difficult for three-component or higher-order systems.

Several years ago, the multi-signal sedimentation velocity (MSSV) approach was introduced [2] as a new tool to address this problem. It takes advantage of the strongly size-dependent migration in the centrifugal field in a configuration that leaves complexes always in a bath of their components, such as to maintain populated complexes in solution during the experiment despite their differential sedimentation velocities. MSSV exploits the relatively high resolution that can be achieved in modern diffusion-deconvoluted sedimentation coefficient distributions [3] and synergistically combines this with spectral deconvolution of absorbance and/or refractive index optical signals [2], [4], [5]. Among the virtues of this method are the relatively fast experimental time, the ability to detect multiple co-existing complexes, the orthogonal observations of composition and complex size and hydrodynamic shape often allowing for an internal test for consistency of the derived complex stoichiometry, and the relative independence of estimates of sample concentrations. Dependent on the particular molecules under study, these often make the MSSV more attractive than other solution methods such as isothermal titration calorimetry, sedimentation equilibrium, or single-signal sedimentation velocity. Many applications of MSSV to two- and three-component systems have demonstrated the power of this approach [6]–[16].

One potential drawback of MSSV is that interacting systems with rapid chemical interconversion on the time-scale of sedimentation will not hydrodynamically resolve the different chemical species during the sedimentation process, but exhibit coupled migration and produce so-called reaction boundaries. This previously limited the application of MSSV to systems that either have slow reaction kinetics (i.e. complex lifetimes on the order of hours) or to conditions where complex formation can be substantially saturated. However, since the original development of MSSV, significant progress has been made in the theory and conceptual understanding of reaction boundaries [17]–[19]. In particular, the effective particle theory (EPT) establishes simple rules for the composition of reaction boundaries [17], opening these for quantitative interpretation with regard to the binding affinity and/or stoichiometry. One objective of the present work was to illustrate how EPT can be applied in the context of MSSV analyses of rapidly interacting systems.

A very useful feature of MSSV is that, due to the high statistical precision of data acquisition, components may be distinguished in the context of significant spectral overlap. In many cases, intrinsic differences in UV absorbance from the content of aromatic amino acids may suffice to resolve components, and extrinsic chromophoric labels may not be necessary [2]. Recently, two of us (C.A.B. and S.B.P.) have developed criteria for the reliability of the MSSV analysis [5], and, based on the component extinction coefficient matrix at the different signals, introduced a quantitative predictive measure for the feasibility of spectral discrimination in MSSV. When spectral discrimination is insufficient, misassignment of signals can occur, with the result of incorrect identification of species' compositions. One of the tell-tales of spectral misassignment is that the total integral over the components sedimentation coefficient distribution does not approximate the known loading concentrations. This observation motivated the question of how the approximate knowledge of total loading concentrations of each component could be used as a constraint to stabilize the MSSV data analysis.

Mass conservation constraints have long been successfully used in the analysis of sedimentation equilibrium of interacting systems [20]–[23], but previously not been used in sedimentation coefficient distribution analyses. In the present work, we describe a novel approach, termed mass conservation constrained multi-signal sedimentation velocity (MC-MSSV), that allows one to introduce approximate total loading concentration either as a strict constraint or as a ‘soft’ regularization parameter. Specifically, we will first show theoretically how mass conservation constraints can achieve a well-conditioned analysis with unambiguous solutions even where the extinction coefficient matrix is singular. Next, we will demonstrate the behavior of MC-MSSV with simulated data, and introduce the combination with constraints in the sedimentation coefficient range of one of the components. Finally, we illustrate the practical application of MC-MSSV on an experimental model system from the interaction of bovine lactoferrin and Tp34 from *Treponema pallidum*.

## Methods

### Theory

#### Multi-signal sedimentation velocity (MSSV)

Briefly, MSSV is a generalization of the *c(s)* method [3] for the global analysis of SV data for macromolecular mixtures acquired at multiple signals [2]. Let us assume we have *m* macromolecular components (1…*M*) with signal coefficients *ε_m_^λ^* at the different signals *λ* (1…Λ, Λ≥*M*) that are used to record the evolution of the sedimentation process, producing data points *a_r,t_^λ^* at radius *r* and time *t*. Values for *ε_m_^λ^* are typically extinction coefficients when considering absorbance data, but could equally represent molar signal increments, for example, for interference data, or more generally any other processes contributing to the signal proportionally to the molar concentration, such as fluorescence, in the absence of non-linearities in the detection arising from inner filter effects or scattering. However, for the efficiency of notation, we use the same symbol *ε_m_^λ^* and the term “extinction coefficients” for the purpose of this theoretical treatment. The sedimentation process is described as a superposition of normalized Lamm equation solutions *L_s,r,t_^(λ)^* [24], [25] of ideally sedimenting species at a range of sedimentation coefficients *s* (1… *N*), and using the customary hydrodynamic scaling law, based on a common frictional ratio, *f_r_*, to estimate the respective diffusion coefficients [3]. Due to unavoidable differences between signals in data-acquisition times radial data points, and sometimes in radial calibration and apparent meniscus positions, in practice the evaluation of the Lamm equation solutions *L_s,r,t_^(λ)^* will also be dependent on the signal to be modeled, to match the available data points *a_r,t_^λ^*. Unknown is the distribution *c_k_(s)* of sedimenting species of class *k* (1…*K*<Λ) where the macromolecular composition of each class is given by a stoichiometry *S_k,m_*, such that the extinction coefficient of each class is 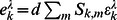 (with *d* denoting the optical path-length). We are searching for the unknown distributions *c_k_(s)* that satisfy the integral equation,
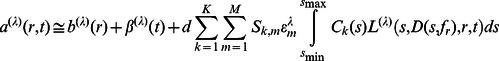
(1)which is approximated by the discretization into a grid of *s*-values, *c_k,s_*, and computed by least-squares,
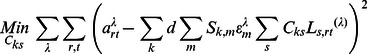
(2)i.e. the minimization of the squared difference between the measured signals and the theoretical contributions of all classes of components to each signal. In Eq. 2, for simplicity the contributions to the different signals from radial-dependent baselines *b^λ^(r)* and time-dependent baselines *β^λ^(t)* (or constant baselines) are not represented, but their inclusion as unknowns to be determined simultaneously with the best-fit distribution trivially follows the algebra outlined elsewhere [26], [27]. Moreover, additional terms from standard Tikhonov-Phillips regularization [3], [28] that are essential for stable solutions are suppressed for clarity. The algebra for solving Eq. 2 with regularization follows the single-signal strategy described previously [3], [29] and is described in more detail below for the case including mass conservation regularization. Modifications of the standard Tikhonov-Phillips regularization for Bayesian regularization [30] were implemented into the MSSV analysis in order to allow one to exploit prior knowledge of the shape of the *c_k_(s)* distributions, such as expected peak positions, but this feature was not used in the present work.

When solving Eq. 2 in the standard MSSV method, the apparent meniscus position of the solution column (separate for the absorbance and interference system due to unavoidable inconsistencies in the radial calibration) and the frictional ratio *f_r_* can be included as unknowns in a non-linear regression. SEDPHAT provides the flexibility to divide the s-range into different segments in which the sedimenting components can be defined separately with respect to their stoichiometry *S_k,m_*. While this feature is very useful to implement constraints, it does not affect the basic principle of Eq. 1. In addition, discrete species of certain molar masses and *s*-values can be added to the distributions or used in place of distributions, with signal increments that are given either in multiples of basis spectra of the macromolecular components, or entered directly. The latter is useful, for example, to add signals from known species, such as well-characterized free species, or signals from impurities, such as models describing the buffer salt signal contributions to interferometric data at very low *s*-values.

A precondition for Eq. 1 and 2 to have a unique solution is that the spectral contributions of the macromolecular components are distinguishable, i.e., that the extinction coefficient matrix has a non-vanishing determinant, 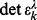
>0. While this condition ensures a mathematical solution, it is not sufficient for the MSSV data analysis in practice. As shown by Padrick & Brautigam [5], a better metric for predicting whether a set of extinction coefficients will be sufficiently different to be distinguishable in the MSSV analysis is the quantity *D_norm_*, defined as
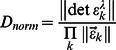
(3)By representing the fractional volume of a parallelepiped whose edges are the vectors of the component extinction coefficients, 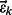, relative to the maximum volume of a hyperrectangle from edges of the same length, it is a measure the spectral ‘orthogonality’ [5]. *D_norm_* values above 0.065 for two-component analyses with dual-signal experiments are desirable for a promising MSSV study. (For for three-component analysis with three-signal experiments, more limited data suggest values >0.01 to be promising.) A *D_norm_* calculator has been implemented in SEDPHAT.

For the practical implementation of MSSV, it is very important to note that sufficiently distinguishable signals with large *D_norm_* values may be obtained for many pairs of macromolecules without requiring extrinsic labels. For example, in some cases, differences in the content of aromatic amino acids and/or in the fraction of carbohydrate moieties can be sufficient for two or three proteins to be distinguishable on the basis of UV absorbance (e.g., at 280 nm and/or 250 nm) and/or refractometric signal increment in the interference optics [2]. In other cases, extrinsic chromophores have been attached to proteins to increase the spectral discrimination.

A statistical problem in the global analysis of signals from different sources, especially when using different optical systems, is that the number of data points as well as the overall signal amplitudes can be very dissimilar. For example, the interference optics provides routinely a higher density of data points than the absorbance system. It can be advantageous to apply corrections to the statistical weights of the different data sets that compensate for the number of data points and/or the signal amplitudes [31].

#### Mass conservation multi-signal sedimentation velocity (MC-MSSV)

The above methodology was extended to make use of estimates of the total molar concentration of each macromolecular component in solution, *C_m_^tot^*. Usually this quantity is not known with complete accuracy but may be estimated from stock concentrations and the pipetting schedule, or better from *c_k_(s)* analyses of single-component samples run in SV experiments side-by-side with the mixture, assuming factors such as adsorption to centerpiece components are similar and co-precipitation of material in the mixture is absent. For this reason, our goal was to implement mass conservation not directly as a hard constraint, but as a scalable regularization term in the form
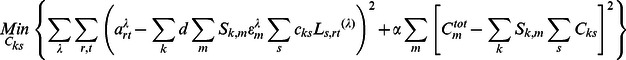
(4)where Eq. 3 is extended by a penalty term that describes the sum of the squared mass deficit for all components. The scaling parameter α can be iteratively adjusted in two different ways: (1) In a ‘soft’ mass conservation approach, it can be adjusted such that the quality of fit to the raw data degrades by no more than a statistically indistinguishable level, pre-calculated by F-statistics. This is similar to the standard regularization [3], [29], and results in the *c_k_(s)* distributions that, among all possible distributions that fit the data, is closest to preserving the total mass. Vice versa, any remaining mass differences result from significant features of the distribution. (2) Since very large values of α can enforce arbitrarily strict mass conservation, it may be adjusted so that the total mass loss is within a preset range, or related, so that the maximum mass defect for any component is within a preset tolerance *δC_m_^tot^*. The value of *δC_m_^tot^* then reflects our confidence interval on the total component concentration *C_m_^tot^*. Dependent on the data, the cost of honoring mass conservation may be a significantly worse fit. Both approaches are useful tools and both have been implemented in SEDPHAT (“auto adjust by chisq, P” *vs* “auto adjust enforce to within (%)”).

To study the effect of mass conservation regularization in relation to spectral discrimination of components it is of interest to follow the solution of Eq. 4. As a quadratic minimization problem we can as usual obtain a linear equation system by taking partial derivatives with respect to any particular unknown, for example, that of component *κ* at *s*-value *σ*, *c_κσ_*, composed of spectral components as given by the stoichiometry *S_κμ_*. After some rearrangement, this leads to
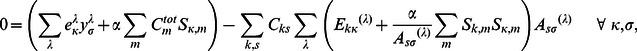
(5)where we use abbreviations analogous to those introduced previously for the sedimentation related vector 
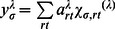
 and matrix 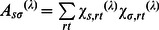 (in vector matrix notation 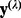 and 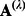) [3], [29] and introduce a matrix of the species' extinction coefficients 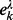 with 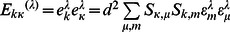 (in vector-matrix notation 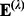). In the single-signal *c(s)* method, Eq. 5 would corresponds to a standard linear system 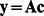 that can be easily solved for non-negative concentrations with standard algebraic methods [3], [29] provided 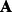 is non-singular. In MSSV without mass conservation constraints (*α* = 0), for a unique solution we rely on the matrix 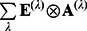 being non-singular. If we simplify the problem by assuming all radial points, time-points, and menisci for the different signals are the same (which is approximately true), then 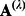 will be independent of signal. For the subset of all *c_k,s_* at the same *s*-value (i.e., *s = σ*), we will then have to rely on the matrix 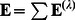 to be non-singular, hence 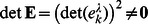 and the above requirement that 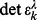 be non-zero. With mass conservation, i.e. for any finite value of *α*, discrimination of species now depends on the non-singularity of the matrix 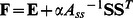, where 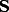 is the stoichiometry matrix, which in the simplest case will the identity matrix 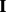. As the determinant is the volume of the parallelepiped, it can be seen that even if 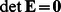 from insufficient spectral resolution, 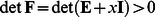 leads to a well-conditioned analysis. It follows that mass conservation constraints can help to overcome a situation in which extinction coefficients are degenerate or close to degenerate. In this sense mass conservation may be regarded as a form of regularization of the spectral dimension of MSSV.

In the implementation in SEDPHAT, an additional refinement was introduced that allows the restriction of the summation range of *c_k,s_* to be considered for mass conservation assessment to a user-defined interval of *s*-values. We also implemented the option that the mass defect of the different components be all weighted on a relative scale instead of an absolute scale (“penalize % defect”), i.e., using 
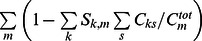
 as an alternative penalty term in Eq. 4. This changes the importance given to mass losses of each component. Finally, the MC-MSSV approach was combined, optionally, with standard Tikhonov-Phillips regularization applied to *c_k_(s)* distributions of each component. In this case, mass conservation regularization goals were ensured for each step during the adjustment of Tikhonov-Phillips regularization.

#### Affinity constant and reaction boundary composition

For reversible binding events that produce complexes with lifetimes on the order of hours or longer, SV will result in the hydrodynamic separation of complex species. These will appear in the diffusion-deconvoluted sedimentation coefficient distributions generally as separate peaks at different *s*-values for different complex species. In MSSV they will produce co-localized peaks in the component *c_k_*(*s*) distributions which represent component concentration contributing to that species. The total of all component *c_k_*(*s*) distributions hence accurately reports on complex compositions. By contrast, for reactions that produce complexes with lifetimes on the order of minutes or less, a sedimentation/reaction process takes place in which the migration of distinct populations of free species of both components and all of the complex(es) are coupled. This process has recently been examined in the effective particle theory (EPT). As a framework for the interpretation of the observed component molar ratios in MSSV for fast reactions, in the following we briefly recapitulate the relationships established by EPT between the reaction boundary composition, the species sedimentation coefficients (*s_A_*, *s_B_*, and *s_AB_* for free A, free B, and the complex AB; the species are assumed to sediment ideally), and the association constant *K* of a 1∶1 reaction.

First, due to the ergodicity of a stable reaction boundary, co-sedimenting populations of the free smaller species must always remain in excess of those of co-sedimenting free populations of the larger species. As a consequence, if we denote with *R_AB_* the molar ratio of total A (the slower sedimenting molecule) to B (the faster sedimenting molecule), then *R* is always less than unity. A second immediate result of EPT is the fact that either free A or free B produce the slower ‘undisturbed’ boundary, migrating with the *s*-value of free A or B, respectively. At a molar excess of loading concentration of the smaller species, the smaller one will always provide the undisturbed boundary. Only if the faster sedimenting species is in molar excess, exceeding a critical concentration for phase transition, c_Btot_*, which for 1∶1 interactions is at
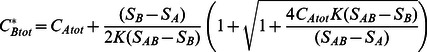
(6)(with *c_Atot_* and *c_Btot_* the total loading concentrations, respectively), then free B can supply the undisturbed boundary and all of A is entirely contained in the reaction boundary [17]. It should be noted that this phase transition occurs close to the equimolar point if the two free components are of similar size, but requires a higher concentration of B for more dissimilarly sized molecules.

For simplicity, assuming a 1∶1 reaction with the smaller species A providing the undisturbed boundary, the sedimentation velocity of the reaction boundary is described by
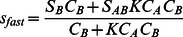
(7)and the composition of the reaction boundary follows
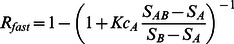
(8)
[17]. It can be discerned from the latter expression that the reaction boundary composition will always be 1.0, i.e. reflecting the complex stoichiometry, if the sedimentation coefficients of the free species *s_A_* and *s_B_* are equal. *R_AB_* will be lower than the complex stoichiometry by a larger margin for more dissimilar *s_A_* and *s_B_*.

Given an experimental value of the reaction boundary stoichiometry and species *s*-values, it is possible to estimate directly the binding constant as
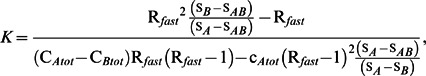
(9)again for the case if the slower sedimenting component constitutes the undisturbed boundary.

### Experimental

#### Protein preparation

Recombinant Tp34 was overexpressed in *E. coli* and prepared as described [11]. The preparation of bovine lactoferrin (bLF; Sigma Chemical Corp.) was also described before [11]. Both proteins were stored in Buffer A (20 mM HEPES pH 7.5, 100 mM NaCl, 2 mM n-octyl-β-D-glucopyranoside) at 4°C.

#### UV extinction coefficients

The method of Pace [32] was used to determine ε_280_ for bLF and Tp34. Briefly, bLF was denatured in 6 M guanidinium hydrochloride, and its absorbance at 280 nm was determined. The extinction coefficient of the protein under these conditions was taken to be the weighted sum of the coefficients of the chromophoric amino acids in its primary sequence. With this knowledge, the concentration of the denatured protein could be calculated. The absorbance of an identical solution under non-denaturing conditions was then obtained; because the concentration of this sample was known, its extinction coefficient could be calculated. This coefficient was used for all experiments. The ε_280_ of Tp34 was determined in the same way.

#### Analytical ultracentrifugation

Prior to centrifugation, bLF and Tp34 were diluted from their stock solutions into Buffer B (20 mM Tris pH 7.5 and 20 mM NaCl). To maintain a compositional balance between the sample and reference sectors, references were prepared in parallel by diluting Buffer A into Buffer B in amounts that mimicked the protein-containing samples. Three samples were prepared: one containing only 13.8 µM Tp34, another containing only 4.6 µM bLF, and the third having a mixture comprising 6.9 µM Tp34 and 2.3 µM bLF. The concentrations of the first two samples were chosen to give convenient pipetting volumes during their preparation. Although not necessary, we sometimes plan the experiment such that concentrations of the components alone are exactly the same as those in the mixture. This strategy allows the experimenter to insert the concentration values derived the analyses of the components alone directly into the mass-conservation calculations (see below). In this case, we used exactly half of the concentration of the proteins in the mixture, another computationally convenient approach. The samples and references were placed in the respective sectors of dual-sectored Epon centerpieces; each centerpiece had been sandwiched between two sapphire windows. Three assembled centrifugation cells (one containing the Tp34 alone, another containing the bLF alone, and the third containing the Tp34/bLF mixture) were inserted into an An60-Ti rotor, which was placed in a Beckman Optima XL-I analytical ultracentrifuge (Beckman-Coulter) and allowed to equilibrate to the experimental temperature (20°C) under vacuum for approximately 1.5 hours. After that, centrifugation was commenced at 50,000 rpm and continued until both proteins had completely sedimented. Data were acquired either in combination of interferometry with absorbance at 280 nm, or in combination of absorbance 250 nm and 280 nm. In all cases, the absorbance optical system was set to scan in continuous mode, with a radial resolution of 0.003 cm. These are our standard settings for all SV experiments; they offer an excellent balance of scanning speed and radial resolution. There is no need to accelerate radial scans in MSSV detection over the standard settings when using absorbance data acquisition at a single wavelength, as the frequency of measuring the boundary position is not different in MSSV from that of standard SV experiments, even though data are acquired sequentially at different signals. The use of wavelength scans in the centrifuge prior to sedimentation, although reporting on relative extinction coefficients at different wavelengths, does not provide sufficiently precise data for use in determining the mass constraints or extinction coefficients in the context of MSSV. Instead, the analytical strategy outlined in the Results section is used.

#### Data analyses

SEDPHAT version 10.31 was used for all of the analyses of the experimental (i.e. non-simulated) data. The buffer density and viscosity were estimated using SEDNTERP [33]. However, we chose to fix the partial specific volumes of the proteins at 0.73 mL/g. This action has the advantage of giving a common *s*_20,*w*_ grid for all data presented, with the drawback of small inaccuracies in representing the frictional ratios, *s*_20,*w*_ values, and masses of the proteins, although compensatory corrections could be easily applied. Unless otherwise mentioned, all analyses had two “spectra” per segment of *s*_20,*w*_-space. Spectrum 1 corresponded to the *c_k_*(*s*) distribution accounting for Tp34, and Spectrum 2 was the *c_k_*(*s*) distribution accounting for bLF. The “low-*s*” constraint (see Results) was achieved by allowing only one *c_k_*(*s*) distribution (i.e. Spectrum 1) in the low-*s* segment of *s*_20,*w*_-space. In all cases, Tikhonov-Phillips regularization was used with a “P-level” of 0.7. The larger number of data points in the interference data compared to the absorbance data results in a larger statistical weighting of the former in the refinement of parameters. A compensatory factor has been introduced into SEDPHAT that ensures the equal weighting of the data sets [4],[31]. However, when we employed these compensations to the data presented in this report, we found only very small differences in reported molar ratios (∼5%) compared to the unweighted analyses; we present the latter in this paper. Indeed, there is a sound reason to allow the interference data to have a slightly higher statistical weight in these analyses: an unexplained and irreproducible optical artifact in the cell containing the mixture of the two proteins is present only in the absorbance data (see Results and Figure S7). Thus, the optimal weighting of the data sets will generally depend more on systematic than statistical errors of the experiment. Generally, it is a good practice to ensure the independence of the results on the weighting procedure applied [31]. A detailed protocol for the analysis of MSSV data has already been supplied in the Supplemental Information of [5]. We have included as supplemental information to this paper an addendum to this protocol that describes the additional parameters that must be input for a mass-constrained MSSV analysis (Supporting Material S1). Plots of the signal profiles, fits, and residuals, as well as the MSSV results were created using GUSSI (biophysics.swmed.edu/MBR/software.html).

## Results

### Computer simulations

As a first test of the MC-MSSV analysis approach, sedimentation profiles were simulated for a 100 kg/mol, 6S-protein ‘B’ (ε_IF_ = 275,000 M^−1^ cm^−1^ and ε_280_ = 100,000 M^−1^ cm^−1^) binding a 20 kg/mol, 2 S-protein ‘A’ (ε_IF_ = 55,000 M^−1^ cm^−1^) with different absorbance extinction coefficients ε_280_ = 20,630, 23,180, or 26,500 M^−1^ cm^−1^ corresponding to the different *D_norm_* values 0.01, 0.05 and 0.10, respectively, creating a 7 S complex with *K_d_* of 2 µM and *k_off_* = 10^−2^/sec (System 1). As can be predicted with the EPT calculator in SEDPHAT [19], at loading concentrations of 20.0 µM A and 5.0 µM B, most of B (4.4 µM) is in the complex, and a reaction boundary at 6.9 S is formed with an apparent molar mass of the effective particle [18] of 117 kg/mol and a composition A/B in the reaction boundary of 0.91. The signal ratios from the reaction and undisturbed boundary are approximately 2∶1, with a total signal of ∼1 OD and ∼3 fringes, respectively (Figure S1). (Similar studies, employing different parameters as suitable for particular experimental systems, can be carried out with the simulation functions of the software SEDFIT and SEDPHAT.)

Due to the relatively small size difference between the complex and the larger component, as well as the potential for different hydrodynamic shapes, the interpretation of the observed *s*-value in terms of complex stoichiometry would be ambiguous. However, the composition of the reaction boundary as observed in an MSSV experiment should give unequivocal information about the 1∶1 stoichiometry.

**Figure 1:**
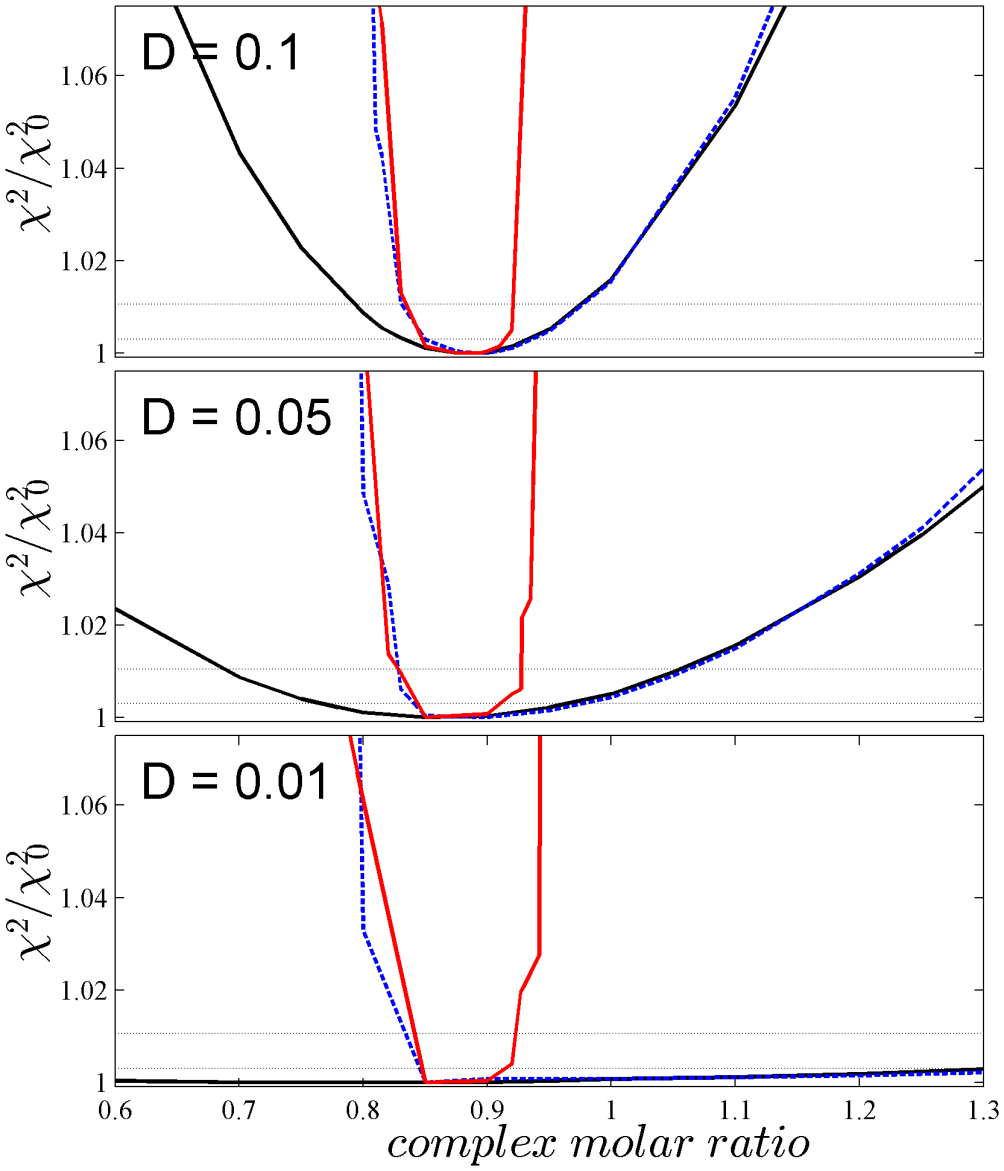
Information content of simulated sedimentation velocity data on the molar ratio in the complex, dependent on spectral properties and MSSV analysis constraints. Data were simulated for a rapid 1∶1 interaction, as described in the Results, with a reaction boundary of composition A/B of 0.91. Raw sedimentation profiles for interference and absorbance detection are shown in Figure S1. Simulated were conditions with acceptable spectral discrimination with *D_norm_* = 0.1 (top), and marginal spectral discrimination with *D_norm_* = 0.05 (middle), and clearly insufficient *D_norm_* = 0.01 (bottom), respectively. The MSSV analysis was conducted with a segmented *c_k_*(*s*) analysis with two different sedimentation coefficient ranges, each describing one of the clearly distinguishable boundaries of the sedimentation pattern: a first segment covering the range of low *s*-values including the *s*-value of the free smaller species and the undisturbed boundary, and a second segment covering the range of sedimentation coefficients of the larger free species and the complex (both of which are entirely engulfed in the reaction boundary of coupled co-transport, which forms a single *c_k_*(*s*) peak in this segment). To probe the statistical accuracy of the molar ratio values in the MSSV analysis, the high-*s* segment allowed only for a single class of species with pre-constrained molar ratio. Plotted is the quality of fit, quantified as the ratio of the χ^2^ obtained and the best-fit χ^2^_0_, as a function of the molar ratio of the high-*s* segment. For each *D_norm_* value, shown are the results from a standard MSSV analysis (black solid line), the MC-MSSV analysis (blue dotted line), and the MC-MSSV additionally constrained by the absence of component B from the low-*s* segment (solid red line). For orientation, the χ^2^/χ^2^_0_ levels corresponding to *P*-values of one standard deviation (at 1.003) or two standard deviations (at 1.011) by *F*-statistics are indicated by thin dotted horizontal lines.

To assess how well the molar ratio can be defined by the data, we probed the change in the quality of fit that occurred when the *c_k_*(*s*) distribution in the range of the sedimentation coefficient of the complex was described by only a single class of species of a composition pre-constrained to different molar ratio values (Fig. 1). The resulting curves are equivalent to a standard error analysis with the projection method and can be subjected to F-statistics [34], [35]. In the absence of mass conservation (Fig. 1, black line), one can discern a significant degradation of the information content on the complex molar ratio with decreasing *D_norm_* value, vanishing almost completely at *D_norm_* of 0.01. When mass conservation was imposed with a tolerance of 1% (blue dotted line), this resulted in a steep increase in the χ^2^ value of the fit towards underestimates of the A/B ratio in the complex, but not for overestimates of A/B. This is due to the non-negativity constraint inherent in *c_k_*(*s*): An overestimate of A in the high-*s* region can be matched by an underestimate of A in the low-*s* region, compensated for by a matching, partial mis-assignment of A to B in the low-*s* region. This can be achieved at the same expense as any spectral mis-assignment of A and B in the standard MSSV. By contrast, an underestimate of A in the high-*s* region will necessarily lead to an inappropriately high concentration of A in the low-*s* region which cannot be compensated for by positive values for B. This leads to a steep increase the χ^2^ of the fit with MC imposed, based on the very large number of data points that define the signal amplitude of a sedimentation boundary.

To further improve the molar ratio resolution a similar steep increase can be achieved towards high molar ratio values if, in addition to mass conservation, we also enforce the condition that there can be no B in the low-*s* region. This is very plausible and known *a priori* because free B sediments faster than free A, therefore populations of B can be safely excluded in the range of the undisturbed boundary formed by free A. This can be achieved easily in SEDPHAT by using a multi-segmented *c_k_*(*s*) model. As expected, the combination of mass conservation with restrictions of the sedimentation coefficient range of B leads to very well defined molar ratio values (Fig. 1, red line), exhibiting a resolution that is nearly independent of the *D_norm_* value from the paired extinction of A and B.

**Figure 2:**
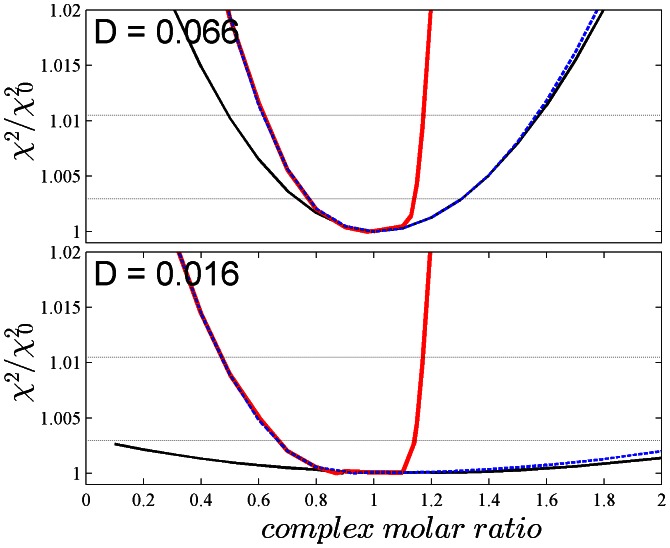
Analogous error surface projections as shown in Fig. 1, but for a more challenging simulated system (System 2) with very dissimilar protein sizes and extinctions (see Results and Figure S2). The data analyses were conducted as described in the legend for Fig. 1, with Tikhonov-Phillips regularization at a level of 0.68. Plotted is again the quality of fit of two data sets with *D_norm_* of 0.066 (Top) and 0.016 (Bottom), quantified as ratio of χ^2^ obtained and the best-fit χ^2^_0_, as a function of the molar ratio of the high-*s* segment. Shown are the error surface projections from a standard MSSV analysis (black solid line), the MC-MSSV analysis (blue dotted line), and the MC-MSSV additionally constrained by the absence of component B from the low-*s* segment (solid red line). For orientation, the χ^2^/χ^2^_0_ levels corresponding to *P*-values of one or two standard deviations by *F*-statistics are indicated by thin dotted horizontal lines.

A second scenario was simulated (System 2) that is more challenging in that, besides small *D_norm_* values, very dissimilar overall signal contributions are present. To this end, the sedimentation of 1.9 µM of a 200 kg/mol, 8.5 S protein B with ε_IF_ = 550,000 M^−1^ cm^−1^ and ε_280_ = 140,850 M^−1^ cm^−1^ was simulated, in a bath of 4.2 µM of a small ligand A of 10 kg/mol, 1.2 S with ε_IF_ = 27,500 M^−1^ cm^−1^ and ε_280_ ranging from 7,300 to 9,000 M^−1^ cm^−1^. The affinity was simulated to be 1 nM and *k_off_* to be 10^−3^/sec, such that virtual saturation of B occurs forming 1∶1 complexes at 9.2 S. Under the simulated conditions, A contributes only ∼5% of the signal of the complex, and the undisturbed boundary composed of free A is only severalfold above the simulated normally distributed noise. To add to the challenge, we simulated a short sedimentation time such that the undisturbed boundary does not yet fully clear the meniscus (Figure S2), and assumed that the precision of MC is only 5%, which at the given total and complex concentrations can propagate to errors in the complex of 17%. Even under these extremely challenging conditions, a similar picture emerges as in the first simulated system: By standard MSSV, the complex molar ratio is well defined at high *D_norm_* but not at very low *D_norm_* values, but the information content can be significantly improved by applying MC constraints that exclude underestimates of the molar ratio, and even more in a multi-segmented *c_k_*(*s*) model combining MC with the *a priori* exclusion of the component with high-*s* monomer from the low-*s* segment, which allows to exclude both under- and over-estimates of the molar ratio (Fig. 2).

In order to verify this picture from the error surface projections independently, we carried out a series of simulations with this System 2, at eight *D_norm_* values and at ten replicate simulations each with independent normally distributed noise. The distribution of best-fit complex molar ratio values as a function of *D_norm_* is shown in Figure S3. Consistent with the above results, we observed large errors and standard deviations of the molar ratio values at low *D_norm_* values, which became strongly reduced when MC was introduced, and further improved in combination with *a priori* constraints in the *s*-range of the larger component.

These simulations also showed that, within the statistical uncertainty of the results, Tikhonov-Phillips regularization can have a side effect of not only providing the most parsimonious *c_k_*(*s*) distributions, but in the process also biasing the molar ratio values (Figure S4). By design, such bias cannot exceed the range of statistically equivalent molar ratio values, which at low *D_norm_* values will be determined by the stringency of the MC constraint (Figure S4).

### Application to the interaction of bovine lactoferrin and Tp34

Earlier [5], a set of four criteria had been established to assess the success of an MSSV analysis. They may be summarized as mass conservation (Criterion 1), distribution rationality (Criterion 2), molar-ratio rationality (Criterion 3), and molar-ratio distinguishability (Criterion 4). We used this framework to assess the performance of MC-MSSV under realistic experimental conditions. We chose bovine lactoferrin (bLF; a ∼80 kg/mol glycoprotein) and Tp34 (∼20 kg/mol) from *Treponema pallidum* as a model system. The interaction of these two proteins had been characterized before using isothermal titration calorimetry [11]. These earlier experiments established that the molar ratio of the interaction is 1∶1, and the calorimetric data were fitted with a 1∶1 binding model; the best-fit association constant was 1.6×10^6^ M^−1^ (*K_d_* = 0.63 µM).

**Figure 3:**
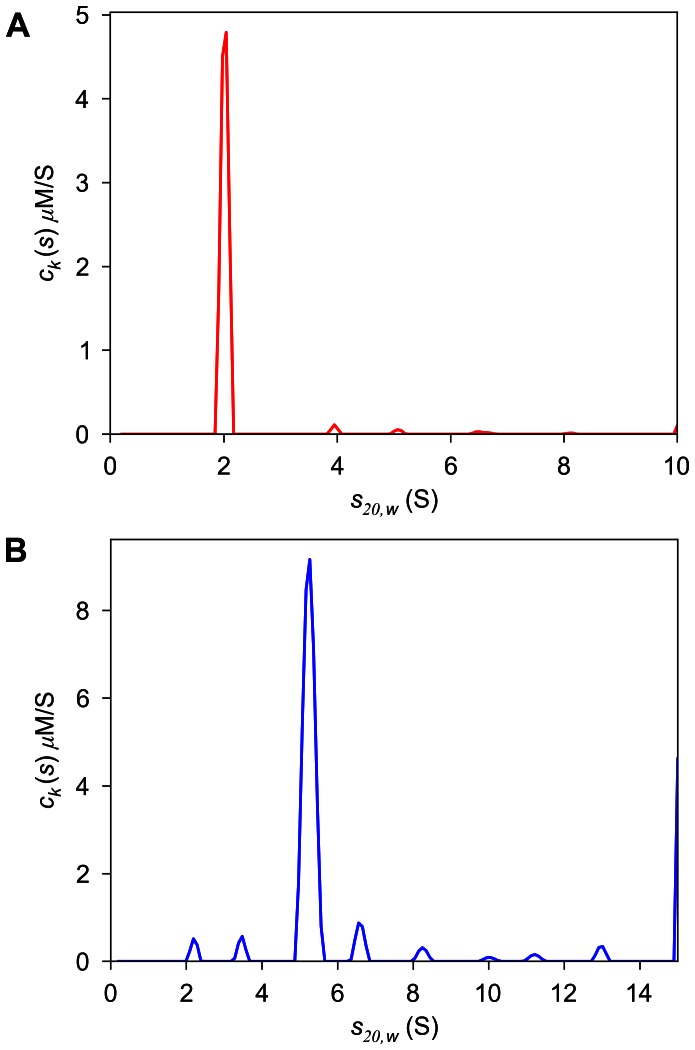
Analysis of the individual samples: *c_k_*(*s*) distributions from the MSSV analysis of SV data of Tp34 alone (A) and bLF alone (B).

First, the spectral properties of the individual proteins were determined. We have generally found that determination of both extinction coefficient using a standalone UV-Vis spectrometer to result in inaccuracies that interfere with the analysis. Instead we recommend choosing one extinction coefficient and using it as a reference for calibrating the others in a preliminary *c_k_*(*s*) analysis on individual components. In many cases, the Raleigh interference signal increment can be estimated very accurately from the polypeptide sequence, but not for substantially modifeid proteins, such as bLF. Instead we used the method of Pace [32] to measure the ε_280_ of bLF and Tp34, which were found to be 108,280 and 31,645 AU×M^−1^×cm^−1^, respectively. Then, SV experiments of bLF alone and Tp34 alone were performed, using absorbance at 280 nm and laser interferometry to simultaneously monitor the evolution of radial concentration profiles of the proteins. The global *c_k_*(*s*) analysis of both signals in terms of a sedimentation coefficient distribution of a single spectral component allows the molar signal increment for the interference signal, ε_IF_, to be determined. For both proteins, this resulted in excellent fits (Figures S5 and S6). The *s*_20,*w*_-value of Tp34 was 2.0 S, and this preparation displays only very small quantities (ca. 4% of the total signal) of aggregated forms (Fig. 3A). The *s*_20,*w*_-value of bLF was 5.2 S, and there is significant evidence of smaller contaminants and aggregated forms of the protein (Fig. 3B). The best-fit values for ε_IF_ were 232,800 and 65,300 fringes×M^−1^×cm^−1^ for bLF and Tp34, respectively. These results indicate that the spectral discrimination between bLF and Tp34 should be poor; the *D_norm_* of this system is 0.016.

**Figure 4:**
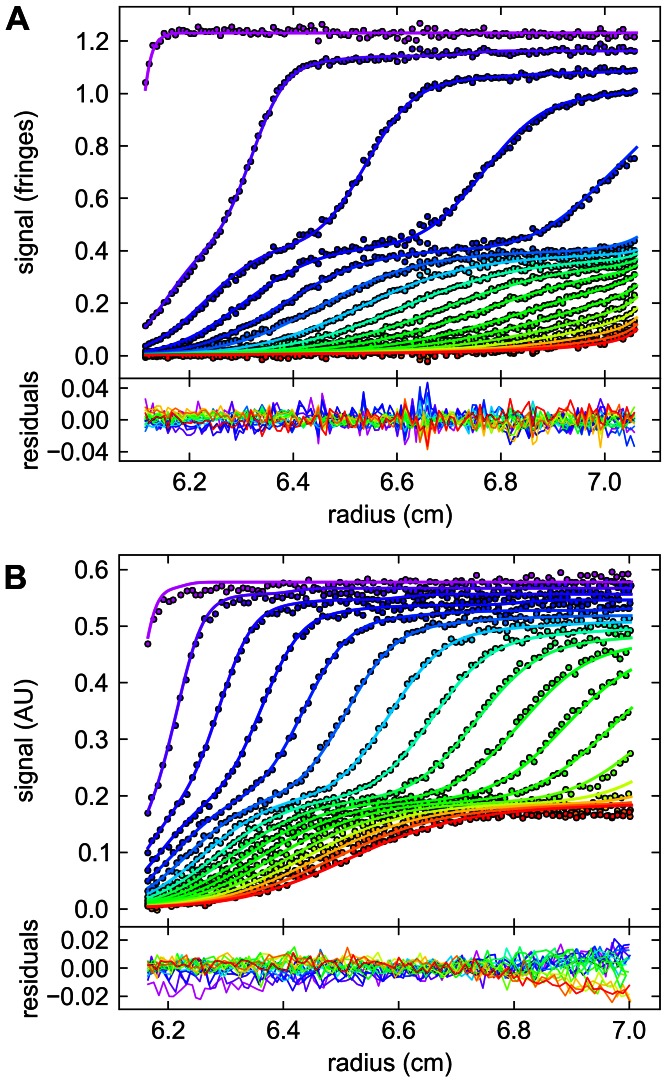
Unconstrained MSSV analysis of the mixture of 6.9 µM Tp34 with 2.3 µM bLF. (A) Interference data (circles), fits (lines) and residuals. Only every 3^rd^ scan and every 10^th^ data point used in the analysis are shown. The time-points of the boundaries are indicated in rainbow colors, progressing from purple (early scans) to red (late scans). (B) Absorbance data, fits, and residuals, showing every 3^rd^ scan and every 4^th^ data point. The best-fit *c_k_*(*s*) distributions are shown in Fig. 5A.

**Figure 5:**
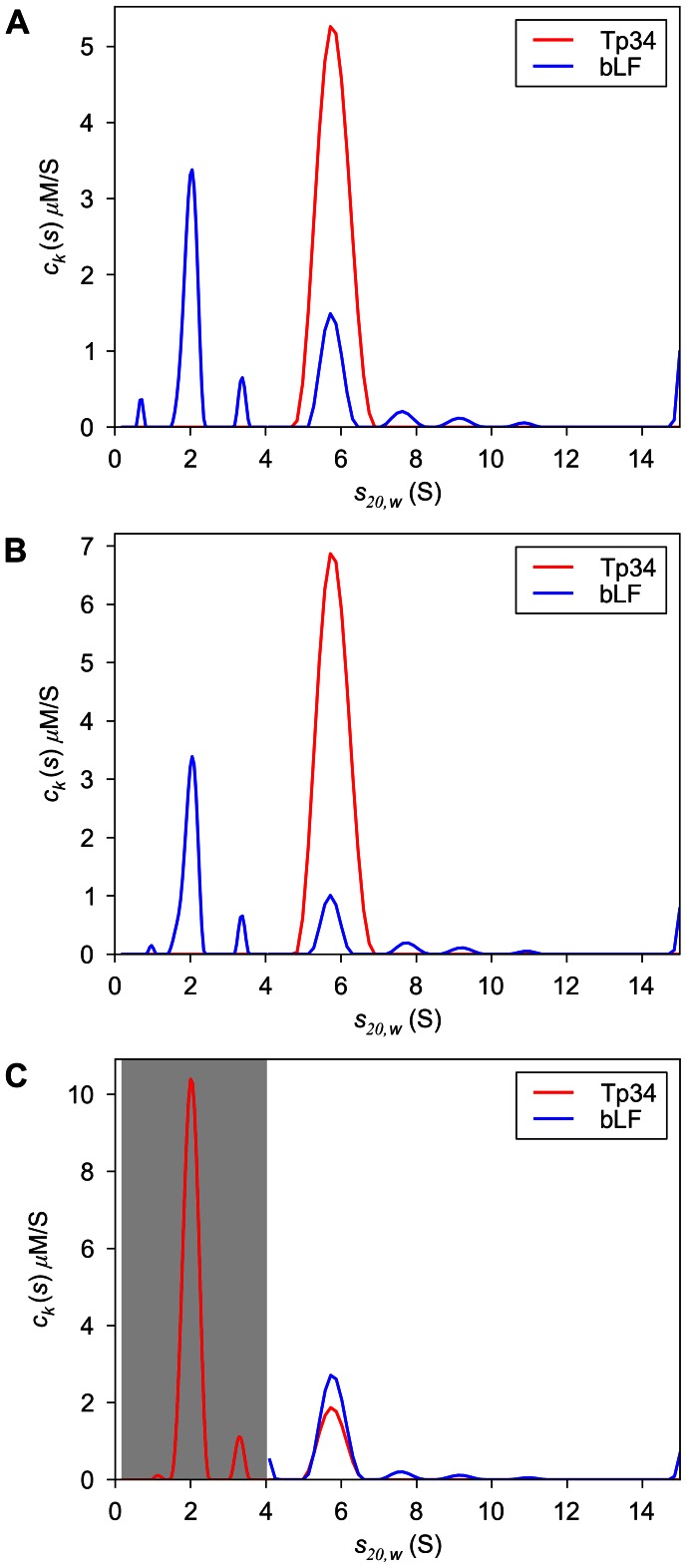
MSSV analysis of the data in Fig. 4. Shown are the *c_k_*(*s*) distributions of both components calculated in a global fit (A) in an unconstrained fit; (B) using mass conservation MSSV; and (C) using both mass conservation and excluding of bLF from the range of sedimentation coefficients between 0 and 4 S (shaded in gray).

Next, an SV experiment containing a mixture of the two proteins was examined. Based on the known pipetted volumes and on integration of the entire distributions in Figures S5 and S6, the concentrations of Tp34 and bLF were 6.9 µM and 2.3 µM, respectively. From the calorimetry results and mass action law we expected that 90% of the bLF would be in a 1∶1 complex having an *s_20,w_* -value >5.2 S. We used standard MSSV without constraints for our first analysis of these data. (These data contained an instrumental artifact that caused a decreasing slope in the absorbance data only at late times and high radial values (not shown); the data were truncated to exclude as much of these artifactual data as possible.) The resulting fits are shown in Fig. 4 and the *c_k_*(*s*) distributions in Fig. 5A. Considering our criteria set out above, the current analysis decisively fails the first three tests: (1) The total concentrations of Tp34 and bLF from integration of the *c_k_*(*s*) distribution were 5.7 µM and 2.8 µM, respectively. These values differ from the known input values by as much as 22%. Given the accuracy of our pipettors and the quality of the technician, we judge that the observed concentrations should vary by no more than 5%, likely much less. (2) Only bLF was detected in the low- *s_20,w_* range, where it should not be found—only Tp34, which is in molar excess, has the size and shape properties to sediment at these low rates. (3) Additionally, the analysis detects a co-sedimenting complex of the two proteins at approximately 5.8 S. However, the molar ratio detected here (4.9 moles Tp34 for 1 mole of bLF) diverges drastically from the calorimetric result. Furthermore, this molar ratio is not supported by the hydrodynamic properties of the complex: such an assembly (5 Tp34's and 1 bLF) at 5.8 S would have a frictional ratio (*f_r_*) of 1.95, whereas the refined frictional ratio of the complex is 1.48. We therefore concluded that the analysis failed due to mis-assignment of the spectral components, which is caused by the lack of sufficiently distinct spectral properties, as predicted by the low *D_norm_* of the system.

In a second analysis, we used mass conservation to constrain the total concentrations of the components to be within 5% of their known values. As shown in Figure S7, an essentially identical quality of fit was achieved. However, again, this strategy failed to satisfy the above criteria: Although the mass conservation succeeded in constraining the total concentrations present over all segments, bLF alone was still detected at low *s_20,w_* –values (Fig. 5B and Figure S7C), which is physically impossible, thus failing Criterion 2. Considering Criterion 3, the ratio of Tp34 to bLF in the complex was even more unrealistic (11.7∶1). Thus, mass conservation constraints alone were not enough to accurately analyze these data.

In a third analysis, in addition to mass conservation, we applied our prior knowledge that only free Tp34 can sediment at low *s*-values. To this end, we divided sedimentation-coefficient space into a low-*s_20,w_* segment (0.2 to 4 S), where only (free) Tp34 was expected, and a high- *s_20,w_* segment (4.1 to 15 S), where both bLF as well as Tp34 were allowed. This doubly constrained model again yields a quality of fit indistinguishable to the original analysis (Figure S8). The resulting *c_k_*(*s*) distributions are shown in Fig. 5C. Because of the newly imposed constraint, only Tp34 is detected between 0.2 and 4 S (gray area of Fig. 5C), and the signal-to-noise ratios of this final analysis are about 130 and 80 for the interference and absorbance data sets, respectively. The cosedimenting complex is observed at 5.8 S, where 1.5 µM Tp34 is found to be complexed with 2.1 µM bLF, yielding a molar ratio of 0.7. This is reasonable based on the known interaction and hydrodynamic properties of this system (see below), thus meeting Criterion 3. For comparison, we performed an analysis with the low-*s* constraint in place but lifting the mass constraint (Figure S9). While the correct concentration of Tp34 is detected at low *s*_20,*w*_-values, the molar ratio in the 5.8-S peak is again drastically incorrect, having a Tp34∶bLF ratio of 4.7∶1 (i.e. a Criterion 3 failure). Thus, both the low-*s* constraint and the mass-conservation constraint are required in this case for a physically meaningful outcome.

It is interesting to note that in the *c_k_*(*s*) distributions of the double constraint MSSV analysis no peak for free bLF was found, yet the complex formation does not seem to be fully stoichiometric. In principle, this could simply be due to a lack of hydrodynamic resolution and regularization of *c(s)* producing a single merged peak. In this case, given the loading concentration and assuming the calorimetric binding constant, the composition of the combined peak would be 0.89. However, no shoulder is evident at 5.2 S in the *c_k_*(*s*) distribution for bLF, and both component distributions appear quite symmetric. This suggests that there really is only one boundary from the coupled migration of free and complex species that occurs when complex lifetimes are short (100 sec or less), as described in the Gilbert-Jenkins [36] and effective particle theories (EPT) [17]. As explained by EPT, in such reaction boundaries the coupled sedimentation of both free and complex species takes place in a way that for fundamental reasons always requires a molar excess of the slower-sedimenting component over the complex stoichiometry [17]. The quantitative prediction of the reaction boundary composition from Eq. 8, based on the calorimetrically binding constant, yields a value of 0.91. Although the observed ratio (0.7) is lower than expected, it should be noted that the association constant on which this calculation was based was measured calorimetric under different buffer conditions, with lower [NaCl] and in the absence of detergent, as will be examined further below. Further, we used F-statistics [35] to examine whether our 0.7 molar ratio was significantly different from 0.9. Using a 68% confidence level, 0.7 is significantly lower than 0.9, but this is not true on a 95% confidence level. Regardless, the outcome is realistic and supports a 1∶1 molar ratio for the complex.

With the first three criteria met in the doubly constrained analysis, we focused on the fourth and examined the statistical significance of the molar ratio value of 0.7. Using the χ^2^ of the fit and F-statistics [35], we chose to test whether varying the molar ratio by 0.5X and 2X would result in a statistically worse fit. Constraining the composition of the high-*s* range to values of either 0.35∶1 or 1.4∶1, while both mass conservation and low-*s* constraints were in place, resulted in significantly (>2σ) worse fits. We therefore judge that Criterion 4 for the success of an MSSV experiment, molar-ratio distinguishability, is also met in this case when applying both mass conservation and low-*s* constraints.

There is an additional fact that supports the reliability of the 0.7 molar ratio detected in the constrained MC-MSSV experiment. To confirm the analysis shown above, we analyzed a follow-up experiment that was conducted almost identically to that described above. However, no interferometric signal was acquired in this second experiment; instead, two UV wavelengths (280 nm and 250 nm) were used to monitor the sedimentation. From the SV experiments of both proteins separately, ε_250_ of bLF and Tp34 were found to be 48,800 and 11,300 AU×M^−1^×cm^−1^, respectively, corresponding to a *D_norm_* value of 0.08, which, according to our simulations (see above and [5]), should afford excellent spectral discrimination of Tp34 and bLF. The MSSV analysis without any constraints yields *c_k_*(*s*) distributions (Figure S10) that are remarkably similar to the constrained distributions of the MSSV analysis based on the combination of 280 nm and interference data (Fig. 5C), with a molar ratio of again 0.7∶1. We conclude that the constraints we placed on the analysis of the IF/ABS280 experiment allowed the MSSV algorithm to perform as if the spectral discrimination of the proteins were excellent.

Having confirmed the measured molar ratio of 0.7 of the reaction boundary, based on knowledge that bLF and Tp34 form 1∶1 complexes, we can use effective particle theory to obtain an independent estimate of the association solely from the observed molar ratio, the known loading concentrations, and the measured or estimated *s*-values of all species. As outlined in the theory section, at a molar excess of the smaller component we are in all cases below the phase transition line such that the conditions of Eq. 9 are fulfilled. Assuming a value of *s_AB_* = 6.2 S (which is not directly measured), then the measured molar ratio of 0.7 results in an estimate of *K_d_* = 3.1 µM under the present buffer conditions, and a theoretical value for the reaction boundary sedimentation coefficient *s_fast_* = 5.84 S which is consistent with the experimentally observed reaction boundary *s*-value. (To show the sensitivity of *K_d_* estimates to the molar ratio value, for *R_fast_* in the range from 0.6–0.8 we calculate a range of *K_d_* values of 1.7–4.9 µM.)

## Discussion

In the last decade, sedimentation velocity analytical ultracentrifugation has re-emerged as a popular tool for the study of protein interactions, as the introduction of new theoretical and computational data analysis methods has significantly increased the resolution and precision of this approach. Initially, our focus in the development of sedimentation velocity was the spatio-temporal analysis of the evolution of a single signal. More recently we have broadened the analysis to the deconvolution of spectral dimensions, which can offer significant advantages when studying heterogeneous protein interactions.

In such systems, different approaches have been described for the quantitative determination of binding constants: direct fitting with solutions to coupled systems of Lamm equations that embed a certain reaction scheme [37]–[39], and the analysis of the isotherms of boundary patterns (their overall weighted-average *s*-value, reaction boundary *s*-value, and amplitudes of reaction and undisturbed boundaries) [19], [40]. We have explored and implemented in SEDPHAT both approaches, and found that the direct Lamm equation modeling can in some cases provide useful information on kinetic rate constants, whereas the boundary pattern analysis is significantly more tolerant for sample imperfections. In our experience, the latter is increasingly important for the study of interactions between more components involving more binding interfaces. Such multi-component and/or multi-site interactions also exacerbate a more fundamental problem, which is the question of what type of complexes can form. Even though both direct Lamm equation fitting as well as boundary pattern analysis may be applied with different interaction schemes to rule out incorrect interaction models by trial and error, this becomes increasingly impractical for more complicated systems. In this context, the strength of the MSSV analysis is the ability to define, in few experiments, the stoichiometry of the complexes formed. This is often one of the most important, and non-trivial facts that informs on possible reaction pathways and is fundamentally a prerequisite for any thermodynamic analysis. In addition to *c_k_(s)* providing a relatively model-free analysis of co-sedimentation that can guide the formulation of the reaction scheme for a thermodynamic analysis, in conjunction with the effective particle theory [17], [19], it also highlights experimental designs that will be particularly informative on stoichiometries of complex formation, producing data that can be utilized initially for the *c_k_(s)* analysis as well as at a later stage for the analysis of the energetics.

MSSV exploits spectral information, which, together with the boundary velocity and boundary spread, forms a set of three independent sources of information on the composition of complexes by mass, *s*-value, and composition. Within the precision of typical results of boundary analysis, molar mass estimates and considerations of hydrodynamic shape alone will often fail to deliver unambiguous estimates for the complex stoichiometry both for systems with similar-sized components as well as for very dissimilar sized components. If components are spectrally distinguishable, such ambiguity can be resolved by MSSV. Furthermore, we have shown previously that spectral deconvolution can be synergistic to the hydrodynamic separation of complexes by sedimentation coefficient [2], which for compact particles, scales with the 2/3 power of the molar mass. Interestingly, an extension of another hydrodynamic approach, fluorescence correlation spectroscopy, to the simultaneous global analysis of multiple signals, F3CS, has recently been developed and been described to be similarly applicable to define the thermodynamic states of complex systems with ternary complexes [41]. While there appear to be several analogies, there are also important differences with regard to the required sample concentrations, spectral properties, complex life-times, and hydrodynamic resolution.

One important virtue of MSSV is that, dependent on protein amino acid composition (or, more generally, macromolecular UV/VIS extinction properties), it may be applied label-free. Differences in the ratio of aromatic amino acids leading to different extinction profiles can be sufficient for spectral discrimination in sedimentation velocity using the absorbance and/or interference detection. (Unfortunately, only a single wavelength is currently available for the commercial fluorescence detector, which excludes the simultaneous operation of other detectors.) This is despite the fact that the quality of the commercial spectrophotometer in the ultracentrifuge is far lower than that of most common bench-top spectrophotometers. For example, the limited reproducibility in the monochromator control when scanning at alternating absorbance wavelengths exhibited by individual instruments imposes some restrictions in the selection of wavelengths to be near a minimum or maximum of absorbance or at a spike of lamp intensity (e.g.230 nm). Alternatively, sorting of scans according to the actual detection wavelength, which is more precisely measured than controlled, is possible and supported in a SEDFIT utility function. In practice, it should also be noted that wavelength accuracy depends on accurate wavelength calibration and may be different in different ultracentrifuges. This potential limitation can be circumvented by measuring absorbance spectra and locating peak absorbances of all components in the ultracentrifuge used for the MSSV experiment.

The excellent spectral resolution in MSSV ultimately rests on the exquisite precision of measuring sedimentation boundary heights, which is also exploited in the familiar application of sedimentation velocity to determine trace amounts of aggregates [42], where routinely boundaries with signal amplitudes less than the noise of a single data point can be detected and quantitated due to the large number of points (typically on the order of 10^5^) determining boundaries and plateau signals. Thus, in scenarios featuring a small signal-to-noise ratio (e.g. <10), we expect the current methodology to perform well. Likewise, even in cases featuring a large difference in sedimentation coefficients between the free and complexed species, wherein the smaller species is overrepresented in the analysis (e.g., see Figure S7), there are usually ample data to spectrally resolve the underrepresented species (presumably the complex), and there is no requirement for all species to contribute evenly or to the same number of scans (e.g., see Figure S2). It should be noted that the precision of relative signal contributions obtained from global modeling of SV data far exceeds the precision of wavelength scans, which could only give a very coarse estimate of extinction coefficients and would not be suitable in conjunction with MSSV. An analytical methodology similar in principle to MSSV could be applied to data acquired from size-exclusion chromatography (SEC) when the elution profiles are acquired with different in-line detectors, e.g. a UV detector and a refractometer. However, such a strategy would rely on the complex of interest having a long lifetime on the timescale of the chromatography experiment. This limitation is not present in MSSV [2], [4], [5]. It would further require sufficient resolution between the complex and any free species; when performed properly, sedimentation velocity generally has superior resolution to SEC.

In the present work, we were concerned with cases where spectral discrimination, with or without extrinsic chromophores, is too poor for a reliable spectral deconvolution. We have shown that the introduction of additional knowledge of the approximate total concentrations of loaded concentrations can substitute for insufficient spectral discrimination. While mass conservation principles have been very successfully applied in sedimentation equilibrium analyses [20], [21], [23], and in some form are common as hard constraints in direct Lamm equation modeling [37], [39] and boundary structure analyses [19], [40], they are novel in sedimentation coefficient distribution analyses, which are conceptually more related to data transforms.

In the weakest form, mass conservation prior knowledge may be used as a form of spectral regularization, which probes the space of different possible multi-component decompositions and reveals the solution where the calculated total loading concentration of each component after integration of the distribution is closest to that known to be inserted into the experimental mixture. When used in conjunction with Tikhonov-Phillips regularization for parsimony of the sedimentation coefficient domain, the second regularization term poses the problem of how to scale it independently. In the current implementation of SEDPHAT, we decided *ad hoc* for an adjustment to produce the same relative increase in the χ^2^ of the fit as applied to the Tikhonov-Phillips term and predicted by F-statistics on a certain confidence level. Furthermore, we found that Tikhonov-Phillips regularization will invariably be correlated with spectral assignment (as it similarly biases peak areas within the statistically permitted extent [43]). Therefore, in this weak form as purely a regularization term, once mass conservation is combined with Tikhonov regularization it may not be more useful than allowing one to explore the flexibility of the spectral assignment. For a statistically better-defined result, we would recommend its use in the absence of simultaneous Tikhonov-Phillips regularization.

A stronger variant, which we envision to be the predominant use of MC-MSSV, is the requirement that mass conservation be strictly fulfilled within a preset tolerance, the achievement of which governs the scaling of the penalty term independent from Tikhonov-Phillips regularization. In SEDPHAT this is programmed such that the tolerance is determined by the user, derived, for example, from the estimated experimental reproducibility of pipetting, and comparison with single-component experiments conducted side-by-side. As long as protein concentrations are based on signal coefficients determined in these separate sedimentation velocity experiments on single-component solutions, actual loss of material in the mixture seems unlikely. Pathological processes such as co-precipitation or altered surface adsorption of complexes, as compared to individual components, are possible. Some of these may be flagged in a standard MSSV analysis by mass balances of all components to be negative, in contrast to spectral mis-assignment that over-estimates one component at the cost of the other. At present, MSSV analysis also assumes the absence of hypo- or hyper-chromicity at the detection wavelengths, which could potentially affect apparent mass conservation. The absence of significant spectral changes upon binding may be verified in a bench-top spectrophotometer, and, if present, be eliminated from consideration in MSSV by detection at the isosbestic point. Importantly, complications of mass conservation analyses historically familiar from sedimentation equilibrium analyses, including those related to baseline signals and the location of the base of the cell, are not present in sedimentation velocity.

Although mass conservation constraints can stabilize the MSSV analysis even in the limit where spectral discrimination of components is completely absent, in itself it cannot provide information on which sedimentation coefficient the mass of each component is attributed to. As an extreme example, one may even load data from the same signal twice and perform a stable MC-MSSV analysis, but this will not yield reliable information on how each component partitions into two sedimenting species (beyond the limits set by mass conservation and non-negativity of concentrations). The strongest use of MC-MSSV, therefore, is an analysis where mass conservation is combined with additional knowledge that excludes components from certain sedimentation coefficient ranges. Especially when studying components with dissimilar sized free species, this knowledge will be obvious from inspection of the *c_k_(s)* traces of the samples from the individual components. Conceptually, the introduction of this information is akin to ‘fringe counting’ or ‘free pool’ experiments, where the complex stoichiometry is indirectly assessed based on the observed boundary amplitudes of the remaining free species of one component and the complex. As shown in the Results section, exploiting such principles in the context of MC-MSSV analyses can be very powerful. Obviously, even when spectra are distinguishable, the introduction of such robust knowledge is highly desirable, and can be expected to leverage MSSV to study more complex multi-component systems.

Besides the previously developed initial criteria for distribution rationality and molar ratio rationality [5], the success of the MC-MSSV analysis is best studied in the framework of a segmented *c_k_(s)* distribution probing the error surface projections for different molar ratio values pre-constrained in a segment covering the sedimentation coefficient of the complex (or reaction boundary, see below). In the limit of very low spectral distinguishability, by MC-MSSV one can expect only to determine the average composition of all complexes in the relevant segment. Additional systematic errors may occur, for example, due to errors in the signal coefficients on which MSSV is based. In the present experimental work we have used the method of Pace [32]. When the absorbance extinction coefficients are predicted from amino acid composition, reported errors are typically <5%, but errors exceeding 15% may occur [32]. For proteins without post-translational modifications it may be advantageous to measure the extinction coefficients experimentally in multi-signal experiments with refractive index optics and using the refractive index increments as a fixed point instead of the extinction coefficient. The refractive index increment is less composition dependent [44], although the compositional prediction of the refractive index increment may be warranted to achieve better accuracy especially for small proteins [44]. Obviously, the assessment of the complex stoichiometry is greatly facilitated by the fact that a complex can only contain an integral number of subunits, which for small complexes (as apparent chiefly from the *s*-value) often poses very generous tolerances.

However, non-integral (or non-rational) values for the boundary composition can be expected for reaction boundaries of rapidly interacting systems. In fact, as we have shown in the effective particle theory, these reaction boundaries can be understood and quantitatively well approximated as regular sedimentation and diffusion processes from ‘pseudo-particles’ or ‘effective particles’ that are composed of all interacting species transiently co-migrating in non-stoichiometric amounts [17], [18]. As such, they are equally accessible experimentally by MSSV or MC-MSSV as stable boundaries of real physical species. However, in addition to the reaction stoichiometry, the composition and velocity of the reaction boundary will depend, in a simple relationship, on the *s*-values of all species, the loading concentrations, and the affinity constants [17]. In fact, a well-known hallmark of reaction boundaries is a concentration-dependence of the peak *s*-values of *c(s)*, and this similarly holds true for *c_k_(s)*. Most notably, for fundamental reasons, the content of the slower sedimenting component will be concentration-dependent and always be less than stoichiometric. However, when one of the components is in >5 fold molar excess over the stoichiometry, the composition will converge to represent essentially the properties of a complex saturated with the excess component [4], [17].

More quantitatively, we have shown in the present work how the measured reaction boundary stoichiometry can be used, in a back-of-the-envelope calculation, to estimate *K_d_* (and the complex *s*-value). This highlights the possibility for quantitative interpretation of the observed molar ratio in MSSV of reaction boundaries, which can be useful, for example, to determine whether the measured values are reasonable and consistent with a putative binding model. Obviously, for a more precise characterization of the binding constant and the hydrodynamic properties of the complex, a more extensive experimental series with large concentration range would be highly advantageous, such that one could combine information from the isotherms of signal amplitudes of all boundary components at all signals with the isotherms of reaction boundary *s*-values and overall signal average *s*-values into a global EPT isotherm analysis, as described in [19]. The results from a MSSV data analysis such as shown in the present work could also be helpful in designing more extensive experiments, for example, using the tool of the effective particle explorer in SEDPHAT that helps to predict expected boundary patterns, including the phase transition lines, and thereby aids in the design of dilution or titration series of experimentally feasible trajectories in concentration space [19], [22]. Thus, even though the presence of a reaction boundary from fast chemical exchange on the time-scale of sedimentation preempts the hydrodynamic separation of co-existing complex species and the determination of their composition, the boundary pattern and MSSV *c_k_(s)* analysis can, similar to the standard *c(s)* analysis, still be a highly informative approach.

In summary, we have developed a new analytical technique for MSSV, called MC-MSSV, which can compensate for poor spectral resolution of the components of a complex and result in excellent outcomes for the determination of complex stoichiometries. It utilizes our *a priori* knowledge of the component concentrations to constrain the MSSV analysis. Previously [5], we had recommended that the *D_norm_* (Eq. 3) of a two-component system be greater than 0.065 in order to reliably distinguish two components using their spectral differences. Our present work demonstrates that this limit is virtually eliminated in an MC-MSSV analysis. Further, the addition of a constraint limiting a certain (low) *s*-range to have only one component in it is very powerful in this analytical setting. It should be noted, however, that this constraint is only applicable in situations where the respective diffusional envelopes of the species are clearly resolved. This limitation leads to an important experimental consideration: to populate complexes, it is advantageous to have the smallest component (“A” in the systems presented in this report) present in a molar excess so that the maximum difference between the *s*-values of the free material and the complex is realized. In a previous report [4], we recommended that the smallest component be present in a molar excess for different reasons, and the results presented here make that recommendation even more pertinent.

## Supporting Information

Supporting Material S1 **A series of screenshots and graphs that illustrates how to use the MC-MSSV method in the SEDPHAT software.** The relevant SEDPHAT parameter box entries that relate to the MC-MSSV tools described in the present work are explained.
(PDF)

Figure S1 **Simulated data of System 1, which consists of a 100 kg/mol, 6 S-protein ‘B’ (ε_IF_ = 275,000 M^−1^ cm^−1^ and ε_280_ = 100,000 M^−1^ cm^−1^) binding a 20 kg/mol, 2 S-protein ‘A’ (ε_IF_ = 55,000 M^−1^ cm^−1^) with absorbance extinction coefficients ε_280_ = 23,180 M^−1^ cm^−1^ corresponding to** ***D_norm_*** **= 0.05, creating a 7 S complex with** ***K_d_*** **of 2 µM and** ***k_off_*** **= 10^−2^/sec.** Simulated was a sedimentation experiment at 50,000 rpm, in a 12 mm solution column, scanned in time-intervals of 300 sec and radial increments of 0.001 cm, and with 0.005 OD or 0.005 fringes of normally distributed noise. Shown is every 3^rd^ data point of every 3^rd^ scan.
(TIF)

Figure S2 **Simulated data of System 2 consisting of a 200 kg/mol, 8.5 S-protein ‘B’ (ε_IF_ = 550,000 M^−1^ cm^−1^ and ε_280_ = 140,850 M^−1^ cm^−1^) binding a 10 kg/mol, 1.2 S-protein ‘A’ (ε_IF_ = 27,500 M^−1^ cm^−1^) with absorbance extinction coefficients ε_280_ = 8,000 M^−1^ cm^−1^ corresponding to different** ***D_norm_*** **= 0.032, creating a 9.2 S complex with** ***K_d_*** **of 1 nM and** ***k_off_*** **= 10^−3^/sec.** Simulated was a sedimentation experiment at 50,000 rpm, in a 12 mm solution column from 6.0 to 7.2 cm, scanned in time-intervals of 300 sec and radial increments of 0.003 cm and 0.0007 cm for absorbance and interference data, respectively, and with 0.005 OD or 0.005 fringes of normally distributed noise. Shown is every 3^rd^ data point of every 3^rd^ scan.
(TIF)

Figure S3 **Replicate simulations of System 2 (as in Fig. S2) at different low** ***D_norm_*** **values.** Each simulation obtained different stochastic noise and was modeled by standard MSSV (Top), MC-MSSV with 5% tolerance (Middle), or MC-MSSV with 5% tolerance additionally with excluded component B from the low-*s* segment (Bottom). Plotted are the molar ratio values from integration of the complex *c_k_(s)* peak (black circles). Red vertical error bars indicate the mean ± standard deviation from the set of 10 simulations performed at each *D_norm_* value. With a MC tolerance of 5% on both components at 1.9 µM and 4.2 µM total concentrations, if all errors occur in the assignments of components in the *c_k_(s)* peak of the complex, the resulting molar ratio may range from 0.85–1.17, which is indicated as dotted horizontal lines.
(TIF)

Figure S4 **Same as Fig. S3, but calculated with Tikhonov-Phillips regularization at a confidence level of 0.68.**
(TIF)

Figure S5 **MSSV analysis of Tp34 alone.** (A) Interference data, fits, and residuals. (B) Absorbance data at 280 nm, fits, and residuals. (C) The *c_k_*(*s*) distribution. Here, *k*≡Tp34.
(TIF)

Figure S6 **MSSV analysis of bLF alone.** (A) Interference data, fits, and residuals. (B) Absorbance data at 280 nm, fits, and residuals. (C) The *c_k_*(*s*) distribution. Here, *k*≡bLF.
(TIF)

Figure S7 **Mass-constrained MSSV analysis of the Tp34/bLF mixture.** (A) Interference data, fits, and residuals. (B) Absorbance data at 280 nm, fits, and residuals. (C) The *c_k_*(*s*) distributions.
(TIF)

Figure S8 **MSSV analysis of the Tp34/bLF mixture with both low-** ***s*** **and mass-conservation constraints.** (A) Interference data, fits, and residuals. (B) Absorbance data at 280 nm, fits, and residuals. (C) The *c_k_*(*s*) distributions. The region shaded in gray was constrained to contain signal only from Tp34.
(TIF)

Figure S9 **MSSV analysis of the Tp34/bLF mixture with low-** ***s*** **constraint but without and mass-conservation constraints.** (A) Interference data, fits, and residuals. (B) Absorbance data at 280 nm, fits, and residuals. (C) The *c_k_*(*s*) distributions. The region shaded in gray was constrained to contain signal only from Tp34.
(TIF)

Figure S10 **Unconstrained MSSV analysis of the experiment with dual 280 nm/250 nm absorbance data acquisition.** (A) The data, fit, and residuals for data collected at 280 nm. (B) The data, fit, and residuals for data collected at 250 nm. (C) The *c_k_*(*s*) distributions.
(TIF)
