# Supplementary material for: Multi-Signal Sedimentation Velocity Analysis with Mass Conservation for Determining the Stoichiometry of Protein Complexes
Source: PLoS One. 2013 May 16;8(5):e62694. doi: 10.1371/journal.pone.0062694 (PMC3656001; doi:10.1371/journal.pone.0062694)
Supplement: Supporting Material S1 — A series of screenshots and graphs that illustrates how to use the MC-MSSV method in the SEDPHAT software. The relevant SEDPHAT parameter box entries that relate to the MC-MSSV tools described in the present work are explained. (PDF) [file pone.0062694.s001.pdf]

# The Mass Conservation Additions to the Multisignal Sedimentation Velocity Global Parameter Window in SEDPHAT

Chad A. Brautigam

Shae B. Padrick

Peter Schuck

# Standard Mass Conservation

Here, two "spectra" are being used to describe the information between 0.2 and 4 S.

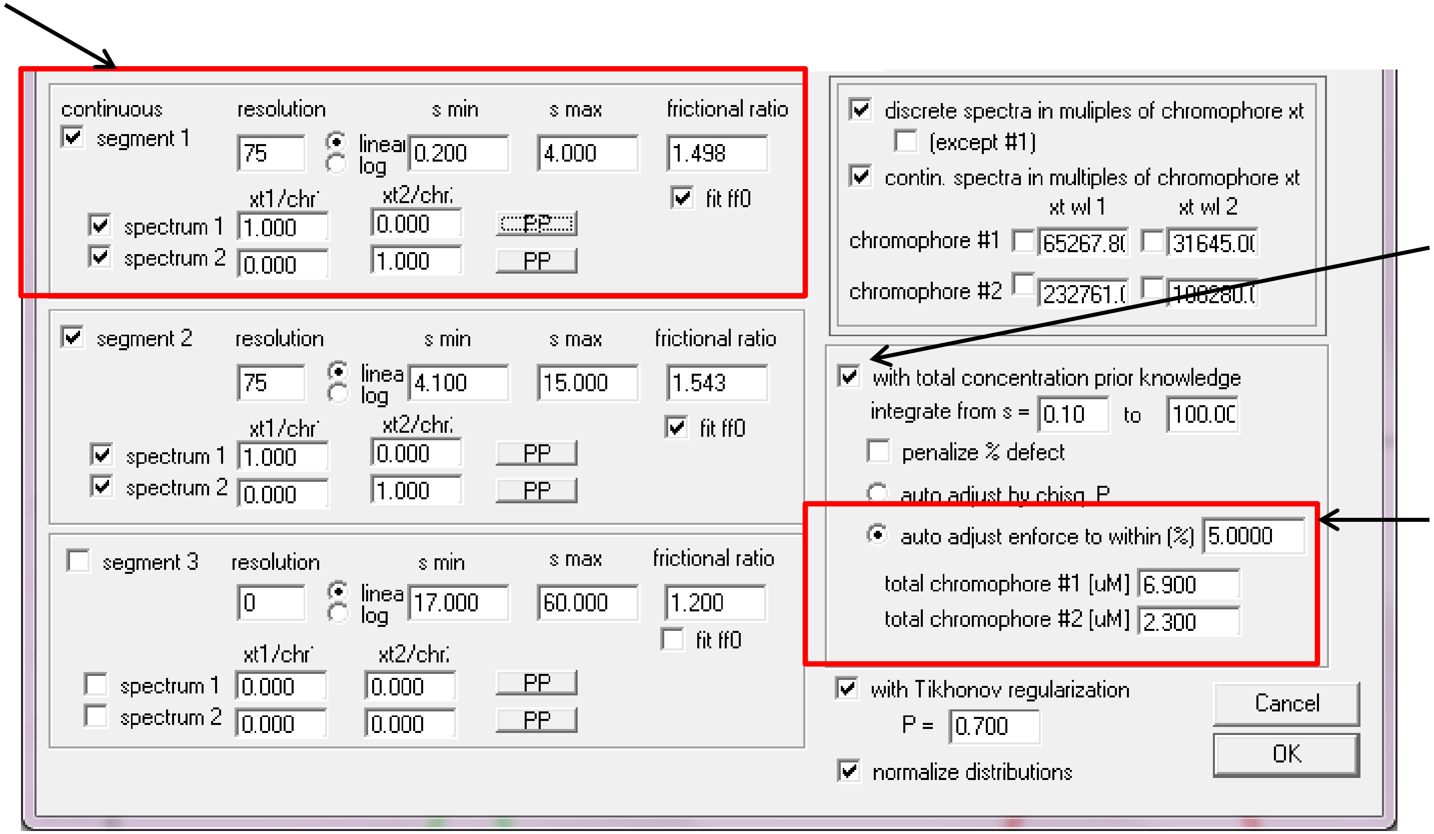


The checkbox turns Mass Conservation on.

The enforce to within % option has been selected, and 5.0 has been input to the entry box. The known concentrations of chromophore #1 (Tp34) and chromophore #2 (bLF) have been input.

# The Resulting Distribution

The preceding parameters could work for a better-behaved case, but there are pathologies present for these data:

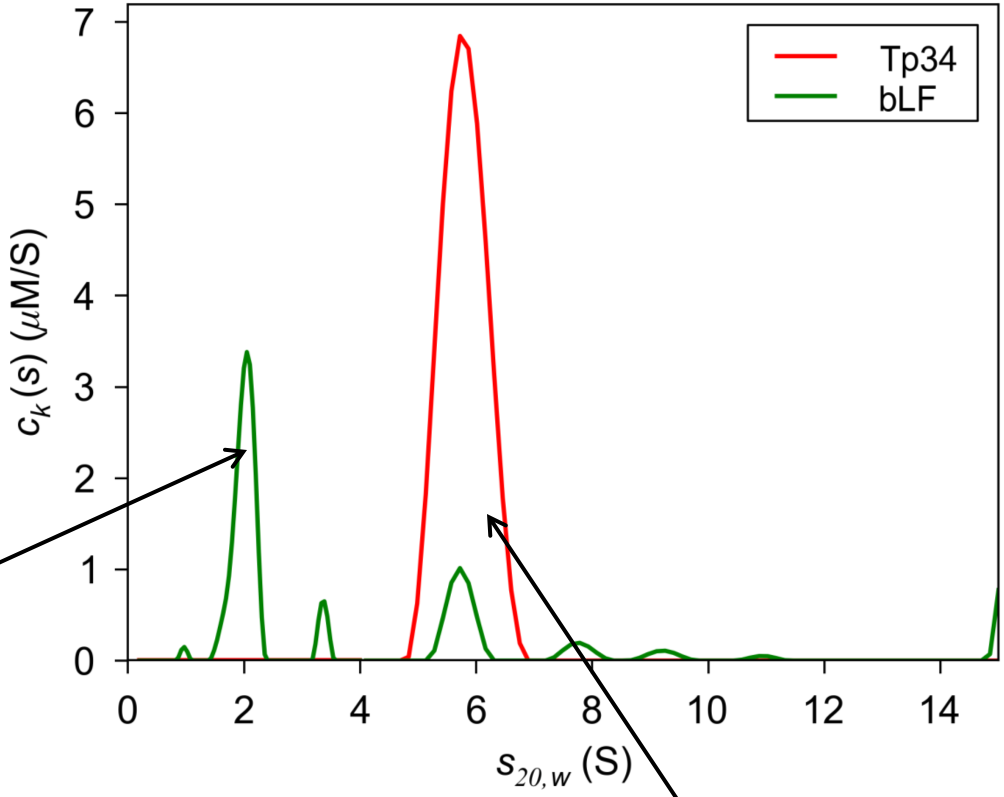


bLF at impossibly low s-values.

Drastically incorrect molar ratio for co-sedimenting material.

# Low-*s*-Constrained Mass Conservation

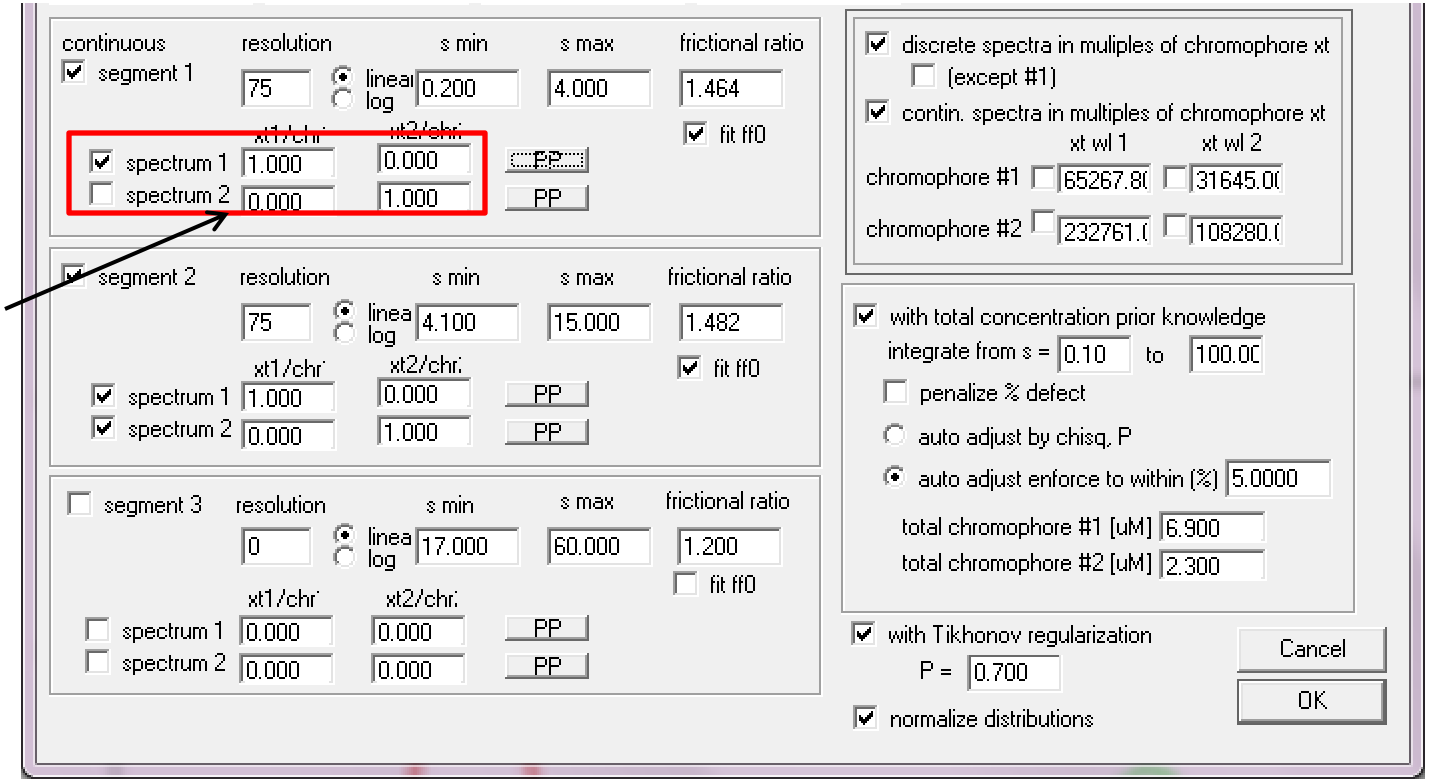


Here, spectrum 2 , which accounts for bLF, has been turned off, forcing all of the low-s material to be Tp34.

# The Final Distribution

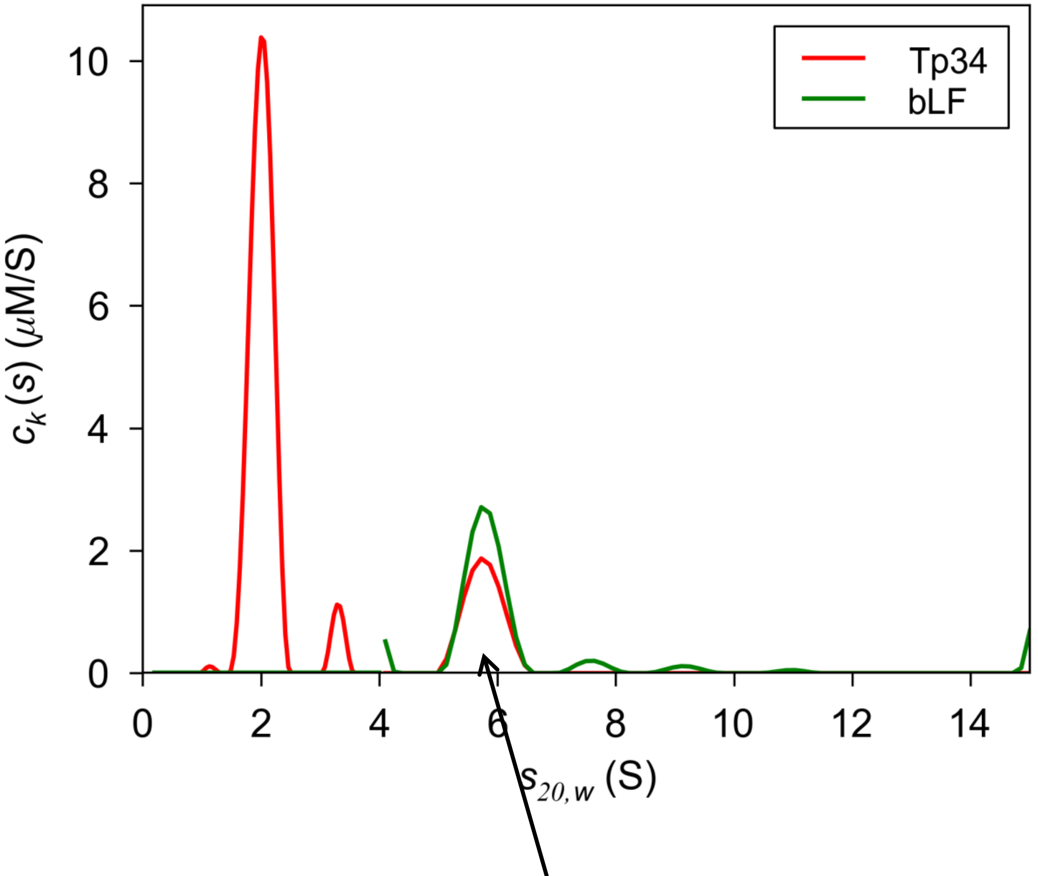


Now, a reasonable ratio of Tp34:bLF is found.
