# Supplementary figures and images for: Multi-Signal Sedimentation Velocity Analysis with Mass Conservation for Determining the Stoichiometry of Protein Complexes
Source: PLoS One. 2013 May 16;8(5):e62694. doi: 10.1371/journal.pone.0062694 (PMC3656001; doi:10.1371/journal.pone.0062694)

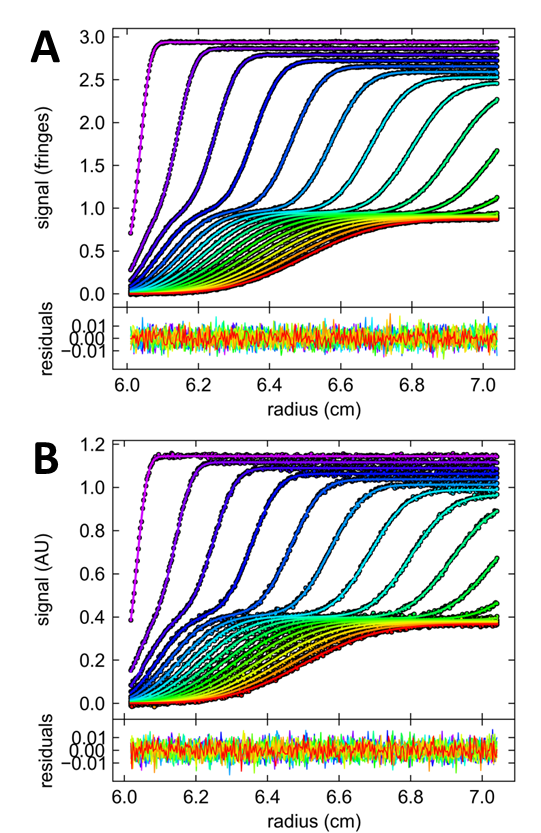

Supplement: Figure S1 — Simulated data of System 1, which consists of a 100 kg/mol, 6 S-protein ‘B’ (εIF = 275,000 M−1 cm−1 and ε280 = 100,000 M−1 cm−1) binding a 20 kg/mol, 2 S-protein ‘A’ (εIF = 55,000 M−1 cm−1) with absorbance extinction coefficients ε280 = 23,180 M−1 cm−1 corresponding to Dnorm = 0.05, creating a 7 S complex with Kd of 2 µM and koff = 10−2/sec. Simulated was a sedimentation experiment at 50,000 rpm, in a 12 mm solution column, scanned in time-intervals of 300 sec and radial increments of 0.001 cm, and with 0.005 OD or 0.005 fringes of normally distributed noise. Shown is every 3rd data point of every 3rd scan. (TIF) [file pone.0062694.s002.tif]

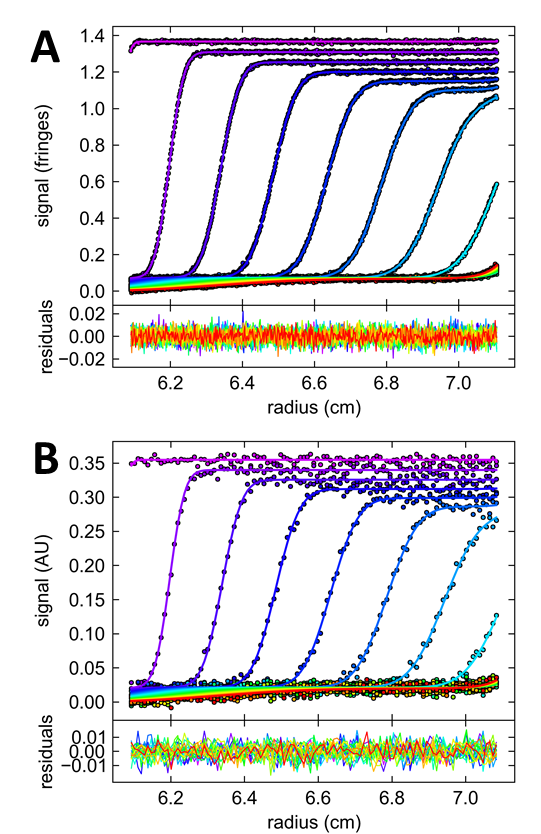

Supplement: Figure S2 — Simulated data of System 2 consisting of a 200 kg/mol, 8.5 S-protein ‘B’ (εIF = 550,000 M−1 cm−1 and ε280 = 140,850 M−1 cm−1) binding a 10 kg/mol, 1.2 S-protein ‘A’ (εIF = 27,500 M−1 cm−1) with absorbance extinction coefficients ε280 = 8,000 M−1 cm−1 corresponding to different Dnorm = 0.032, creating a 9.2 S complex with Kd of 1 nM and koff = 10−3/sec. Simulated was a sedimentation experiment at 50,000 rpm, in a 12 mm solution column from 6.0 to 7.2 cm, scanned in time-intervals of 300 sec and radial increments of 0.003 cm and 0.0007 cm for absorbance and interference data, respectively, and with 0.005 OD or 0.005 fringes of normally distributed noise. Shown is every 3rd data point of every 3rd scan. (TIF) [file pone.0062694.s003.tif]

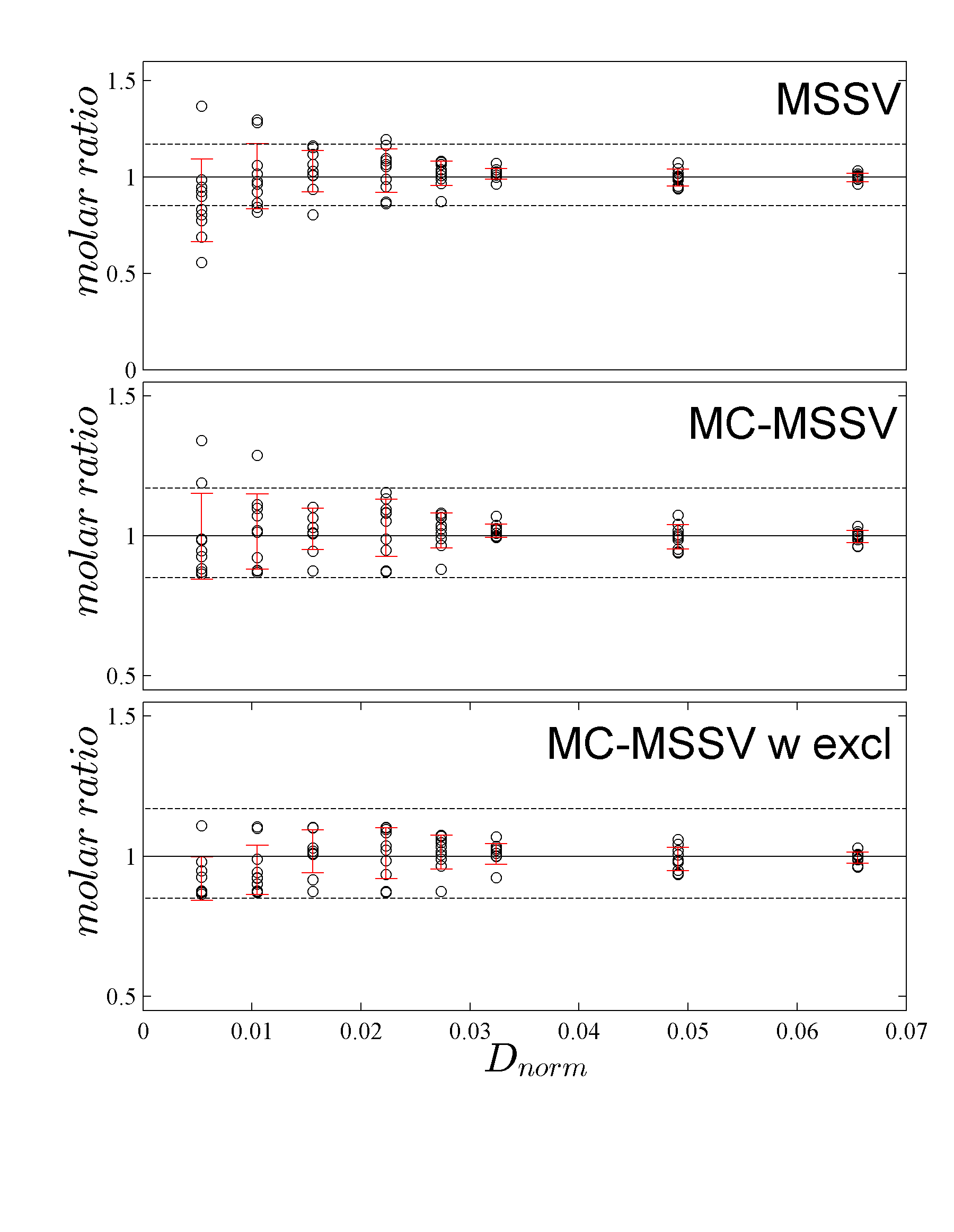

Supplement: Figure S3 — Replicate simulations of System 2 (as in Fig. S2) at different low Dnorm values. Each simulation obtained different stochastic noise and was modeled by standard MSSV (Top), MC-MSSV with 5% tolerance (Middle), or MC-MSSV with 5% tolerance additionally with excluded component B from the low-s segment (Bottom). Plotted are the molar ratio values from integration of the complex ck(s) peak (black circles). Red vertical error bars indicate the mean ± standard deviation from the set of 10 simulations performed at each Dnorm value. With a MC tolerance of 5% on both components at 1.9 µM and 4.2 µM total concentrations, if all errors occur in the assignments of components in the ck(s) peak of the complex, the resulting molar ratio may range from 0.85–1.17, which is indicated as dotted horizontal lines. (TIF) [file pone.0062694.s004.tif]

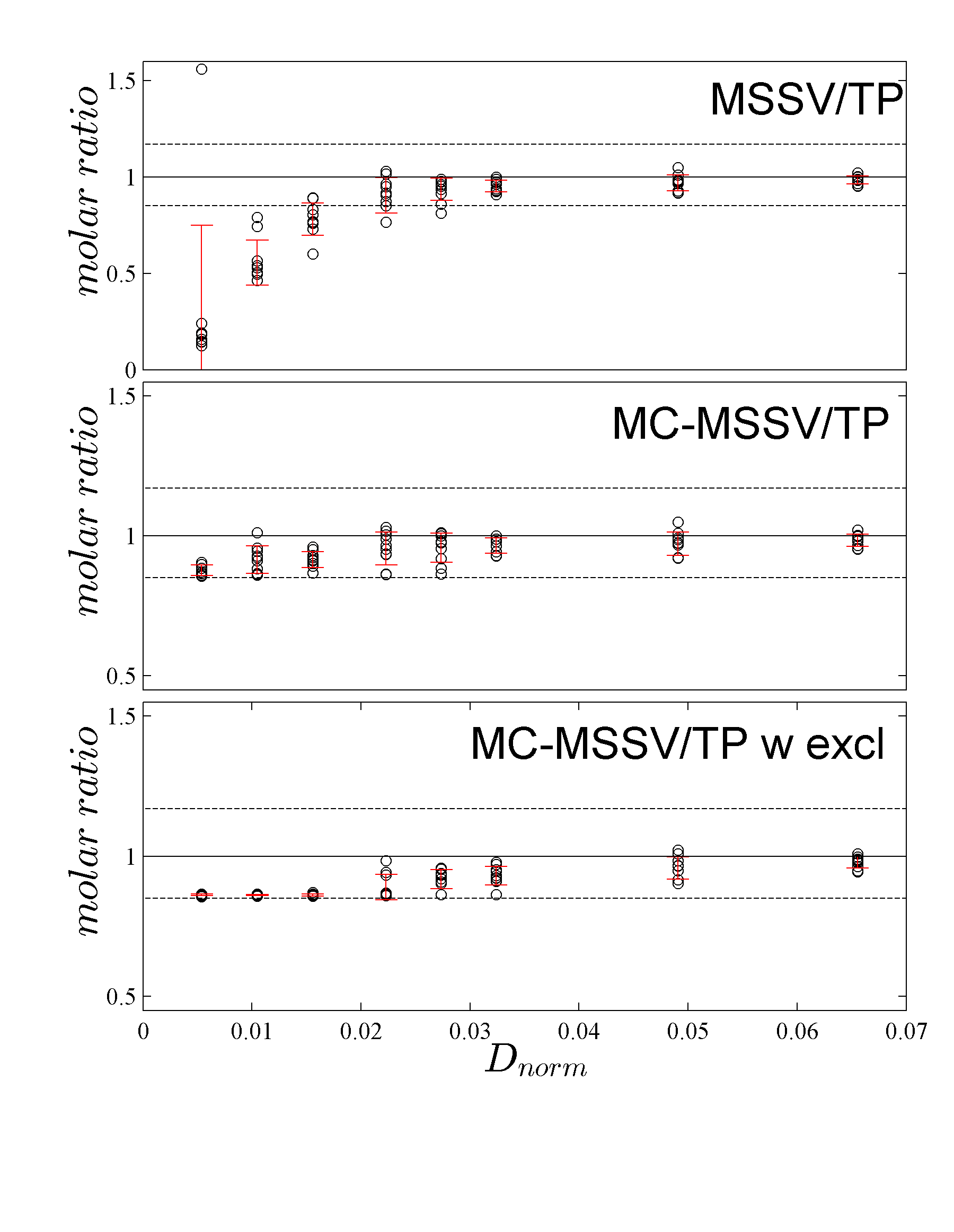

Supplement: Figure S4 — Same as Fig. S3, but calculated with Tikhonov-Phillips regularization at a confidence level of 0.68. (TIF) [file pone.0062694.s005.tif]

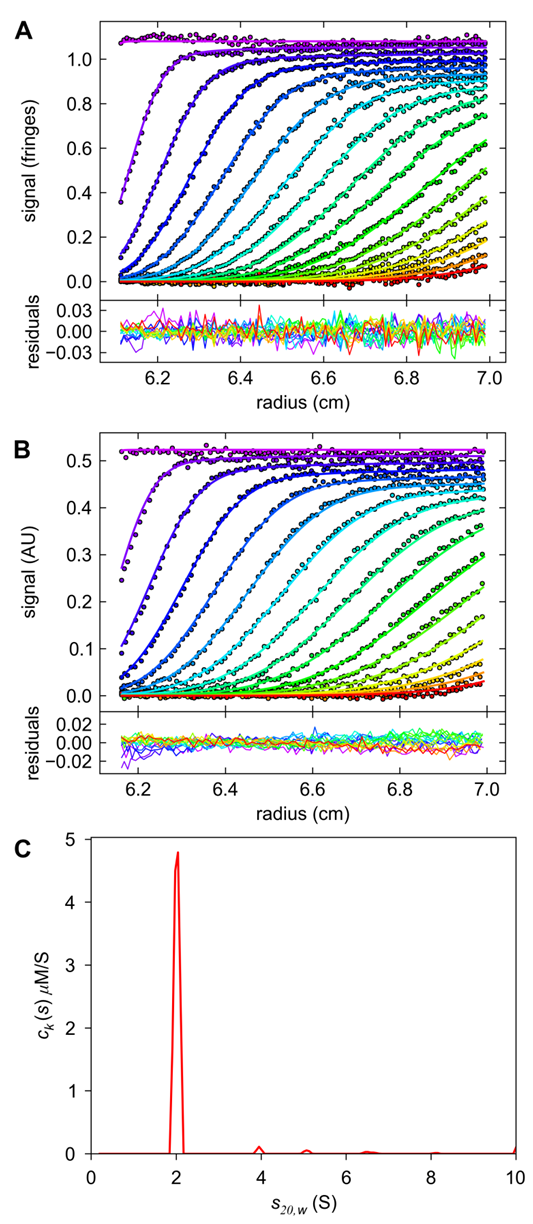

Supplement: Figure S5 — MSSV analysis of Tp34 alone. (A) Interference data, fits, and residuals. (B) Absorbance data at 280 nm, fits, and residuals. (C) The ck(s) distribution. Here, k≡Tp34. (TIF) [file pone.0062694.s006.tif]

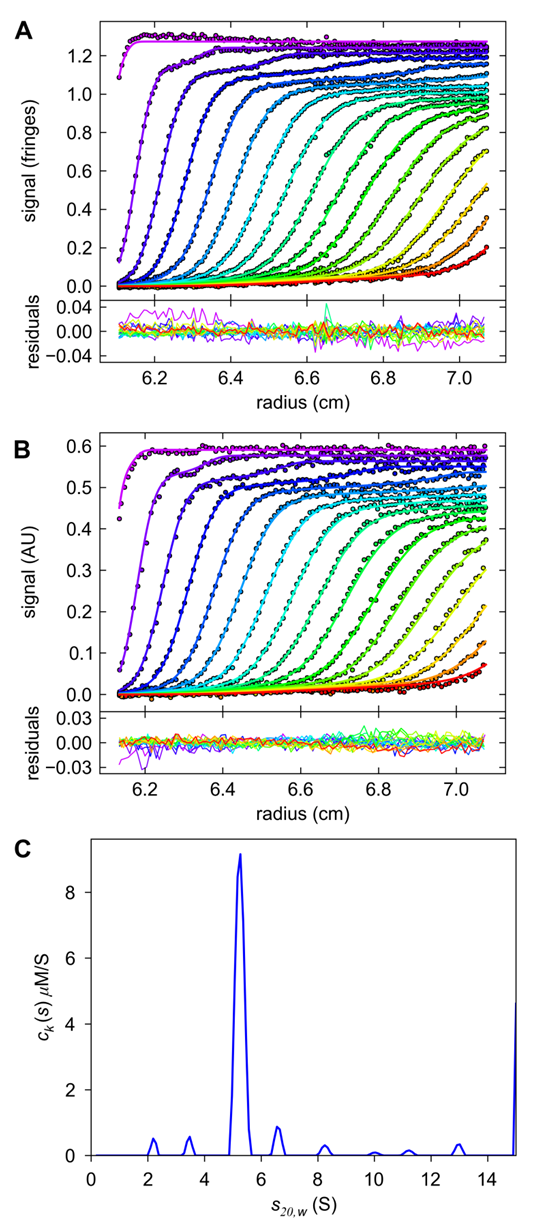

Supplement: Figure S6 — MSSV analysis of bLF alone. (A) Interference data, fits, and residuals. (B) Absorbance data at 280 nm, fits, and residuals. (C) The ck(s) distribution. Here, k≡bLF. (TIF) [file pone.0062694.s007.tif]

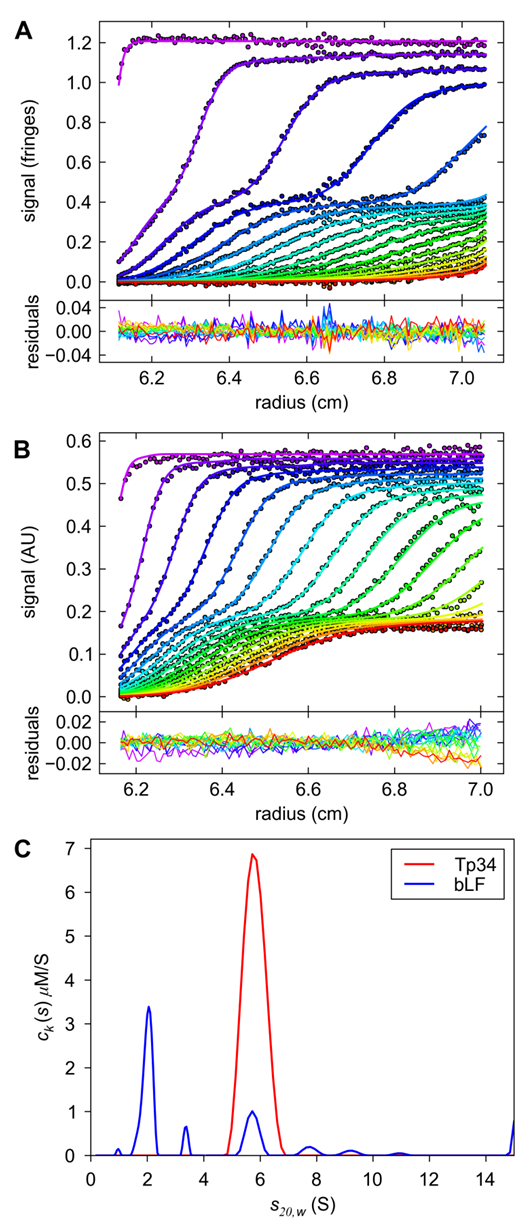

Supplement: Figure S7 — Mass-constrained MSSV analysis of the Tp34/bLF mixture. (A) Interference data, fits, and residuals. (B) Absorbance data at 280 nm, fits, and residuals. (C) The ck(s) distributions. (TIF) [file pone.0062694.s008.tif]

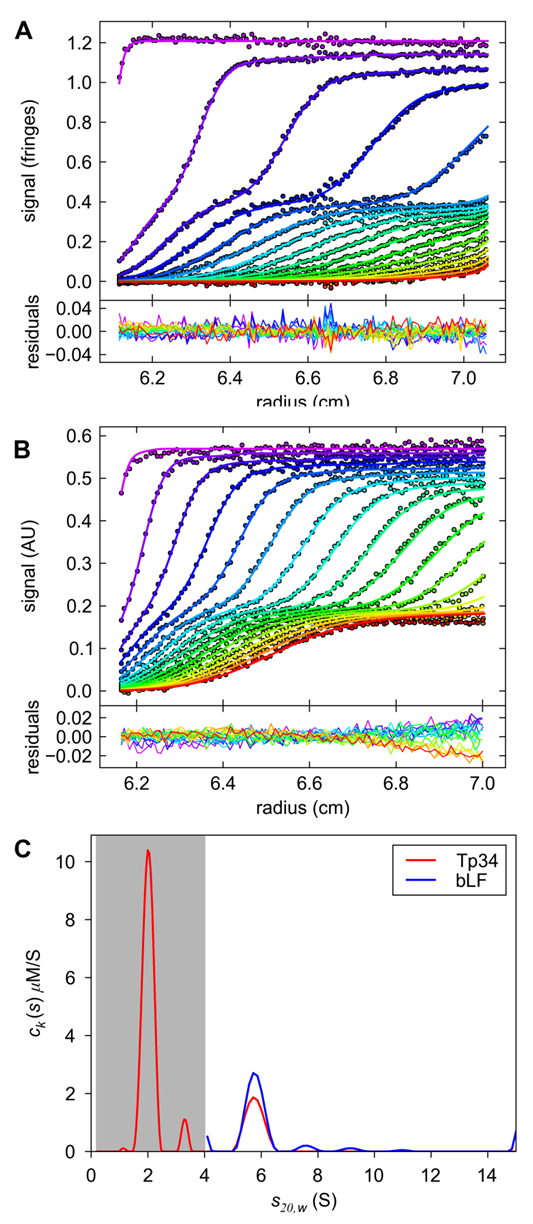

Supplement: Figure S8 — MSSV analysis of the Tp34/bLF mixture with both low- s and mass-conservation constraints. (A) Interference data, fits, and residuals. (B) Absorbance data at 280 nm, fits, and residuals. (C) The ck(s) distributions. The region shaded in gray was constrained to contain signal only from Tp34. (TIF) [file pone.0062694.s009.tif]

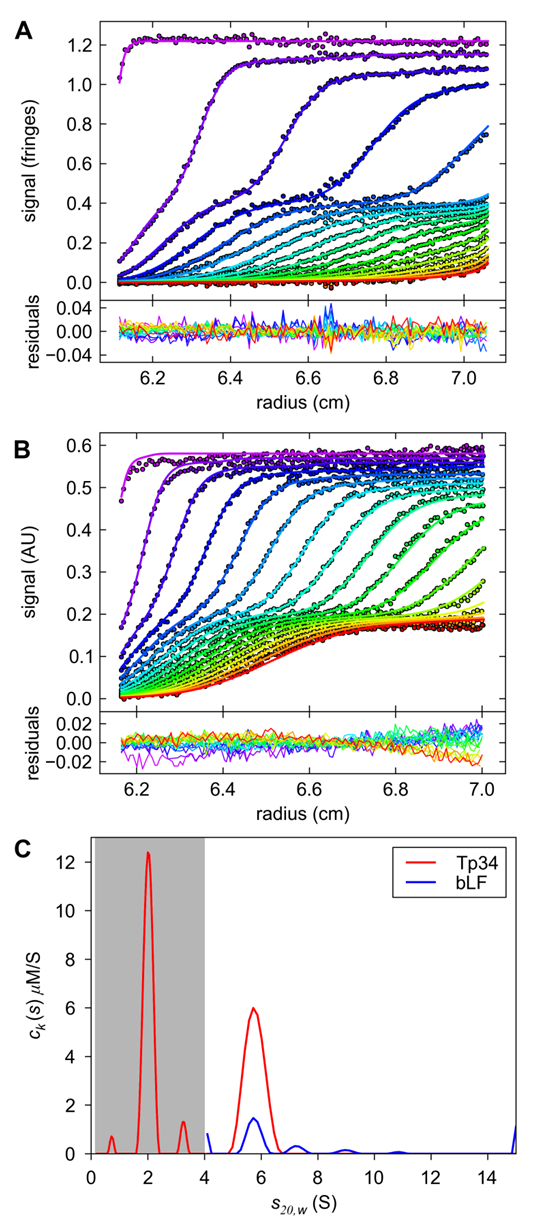

Supplement: Figure S9 — MSSV analysis of the Tp34/bLF mixture with low- s constraint but without and mass-conservation constraints. (A) Interference data, fits, and residuals. (B) Absorbance data at 280 nm, fits, and residuals. (C) The ck(s) distributions. The region shaded in gray was constrained to contain signal only from Tp34. (TIF) [file pone.0062694.s010.tif]

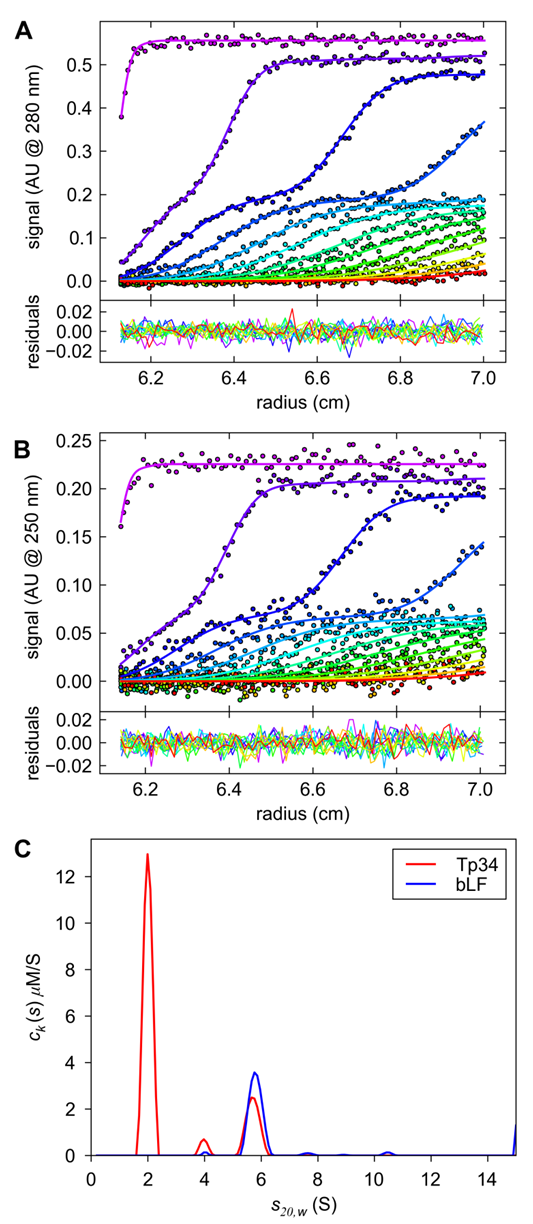

Supplement: Figure S10 — Unconstrained MSSV analysis of the experiment with dual 280 nm/250 nm absorbance data acquisition. (A) The data, fit, and residuals for data collected at 280 nm. (B) The data, fit, and residuals for data collected at 250 nm. (C) The ck(s) distributions. (TIF) [file pone.0062694.s011.tif]
